# Genome-Wide Association Study of *Staphylococcus aureus* Carriage in a Community-Based Sample of Mexican-Americans in Starr County, Texas

**DOI:** 10.1371/journal.pone.0142130

**Published:** 2015-11-16

**Authors:** Eric L. Brown, Jennifer E. Below, Rebecca S. B. Fischer, Heather T. Essigmann, Hao Hu, Chad Huff, D. Ashley Robinson, Lauren E. Petty, David Aguilar, Graeme I. Bell, Craig L. Hanis

**Affiliations:** 1 Center for Infectious Disease, Division of Epidemiology, Human Genetics, and Environmental Sciences, University of Texas Health Science Center, Houston, TX, United States of America; 2 Human Genetics Center, Division of Epidemiology, Human Genetics, and Environmental Sciences, University of Texas Health Science Center at Houston, Houston, TX, United States of America; 3 Department of Epidemiology, The University of Texas MD Anderson Cancer Center, Houston, TX, United States of America; 4 Department of Microbiology, University of Mississippi Medical Center, Jackson, MS, United States of America; 5 Winters Center for Heart Failure Research and Section of Cardiology, Baylor College of Medicine, Houston, TX, United States of America; 6 Departments of Medicine and Human Genetics, The University of Chicago, Chicago, United States of America; University of Iowa Carver College of Medicine, UNITED STATES

## Abstract

*Staphylococcus aureus* is the number one cause of hospital-acquired infections. Understanding host pathogen interactions is paramount to the development of more effective treatment and prevention strategies. Therefore, whole exome sequence and chip-based genotype data were used to conduct rare variant and genome-wide association analyses in a Mexican-American cohort from Starr County, Texas to identify genes and variants associated with *S*. *aureus* nasal carriage. Unlike most studies of *S*. *aureus* that are based on hospitalized populations, this study used a representative community sample. Two nasal swabs were collected from participants (n = 858) 11–17 days apart between October 2009 and December 2013, screened for the presence of *S*. *aureus*, and then classified as either persistent, intermittent, or non-carriers. The chip-based and exome sequence-based single variant association analyses identified 1 genome-wide significant region (*KAT2B*) for intermittent and 11 regions suggestively associated with persistent or intermittent *S*. *aureus* carriage. We also report top findings from gene-based burden analyses of rare functional variation. Notably, we observed marked differences between signals associated with persistent and intermittent carriage. In single variant analyses of persistent carriage, 7 of 9 genes in suggestively associated regions and all 5 top gene-based findings are associated with cell growth or tight junction integrity or are structural constituents of the cytoskeleton, suggesting that variation in genes associated with persistent carriage impact cellular integrity and morphology.

## Introduction

Infectious diseases result from complex interactions between the microorganism, the host, and the environment. Host genetic factors play a major role in determining differential susceptibility to major infectious diseases of humans, including malaria [1], HIV/AIDS [2], tuberculosis [3], hepatitis B [4], Norovirus diarrhea [5], prion disease [6], Cholera [7], and *Helicobacter pylori* infections [8]. The first evidence that genetic factors could impact infectious disease outcomes was derived from epidemiological studies that identified differences between human populations exposed to the same infectious organism [9]. This is equally true for *S*. *aureus* [10–12], but this pathogen represents a special case because it is an opportunistic pathogen that can colonize humans without causing overt disease [13]. It is therefore an ideal system for examining host pathogen interactions.

Even though humans are exposed to *S*. *aureus* at birth, not all are equally susceptible to colonization [9]. Many body sites can be colonized by *S*. *aureus*, but nasal decolonization has been shown to be effective in reducing colonization at other body sites, suggesting that the anterior nares is one of the primary *S*. *aureus* reservoirs [14, 15]. Human carriage has been classified as either persistent, intermittent, or non-carriage with rates of carriage ranging from 10–35%, 20–75%, and 5–70%, respectively, depending on race, age, gender, and whether the population examined was hospital- or community-based [9, 16–18]. Carriage is not representative of infection, *per se*. Rather, carriage impacts the risk of acquiring infection, disease presentation, and disease severity [13]. Furthermore, the genotype of the colonizing *S*. *aureus* strain, the nature of the immune response elicited following exposure, and underlying host genetic factors may all play a role in susceptibility to colonization and/or infection [9, 19–24]. Like other complex conditions, susceptibility to infectious agents does not typically follow a simple Mendelian pattern of inheritance, largely due to the fact that human immune responses are controlled by complex genetic mechanisms and modifying environmental influences [25, 26].

Candidate gene studies have uncovered associations between specific genes and carriage status [20–23, 27–29]. For example, IL4 and C-reactive protein have been shown to be associated with carriage in the Rotterdam Study [20, 22]. In the same study, a 68% reduction in risk of persistent carriage was observed related to the glucocorticoid receptor gene [30] (S1 Table). Polymorphisms in genes encoding different defensins and MBL (manose binding lectin) have also been associated with *S*. *aureus* persistent carriage [20, 31, 32] (S1 Table). The toll-like receptors have also been associated with increased risk of streptococci and enterococci skin and soft tissue infections [21, 33] suggesting that there may be some commonalities in the genetics of susceptibility to infection with different pathogens. No community-based genome-wide association or whole exome sequencing studies have previously been performed in the context of *S*. *aureus* carriage, but recently, 2 hospital-based genome-wide association studies of *S*. *aureus* infections were conducted [34, 35]. That these studies failed to identify targets with genome wide significance is not necessarily surprising since hospital environments themselves are a significant risk factor for acquiring *S*. *aureus* infections and these effects may overwhelm modest genetic influences on risk [36].

The present study was designed to identify genes/markers associated with persistent and intermittent carriage of *S*. *aureus* in a community-based sample of 858 Mexican-Americans from Starr County, Texas. Single nucleotide polymorphism (SNP) data from the Affymetrix Genome-Wide SNPArray 6.0 assay imputed out to the complete SNP set in the 1000 Genomes Project [37] and whole exome sequence data were used to conduct single variant and gene-based burden tests. The single variant analyses identified the *KAT2B* (lysine acetyltransferase 2B) region as significantly associated with intermittent *S*. *aureus* carriage. All 5 top genes identified in the gene-based burden test and at least 1 gene in each region suggestively associated with persistent carriage in the single variant analysis are associated in some fashion with maintenance of cellular integrity, the cytoskeleton, or the cell cycle. On the other hand, genes associated with intermittent carriage were largely associated with immune function, adipogeneisis, or inflammation. These analyses identified little evidence of overlap between genes or regions corresponding to different carriage phenotypes suggesting that each carrier state may be distinct.

## Materials and Methods

### Human subjects

This study and the consenting procedures were approved by the University of Texas Health Science Center Institutional Review Board (HSC-SPH-06-0225). Written informed consent was obtained from all participants before they were enrolled in the study.

### Microbiologic testing

Specimens were collected from the nares using dry, unmoistened sterile BBL^™^ CultureSwabs^™^ Liquid Stuart swabs. Swabs were inserted into the patient’s nostril approximately 1 inch from the edge from the anterior nares placing the swab in proximity with the inferior and middle concha and rolled several times. Bar-coded specimen tubes were stored and shipped at 4°C to the University of Texas Health Science Center at Houston School of Public Health for processing.

To identify and characterize *S*. *aureus* from specimens containing mixed flora, nasal swabs were inoculated on manitol salt agar (MSA) plates (Remel Inc., Lenexa, KS) as described [38]. Following inoculation of primary plates, swabs were broken off into tryptic soy broth for enrichment (TSB) (Remel Inc.). The enrichment broths were vortexed for 10 seconds to ensure that any bacteria still attached to the swab were released into the media and the samples subsequently incubated at 37°C for 48 hours and re-plated on secondary MSA plates. Gram staining of respective colonies that turned MSA plates yellow were used to ensure that selected colonies possessed *S*. *aureus* morphology. Presumptive *S*. *aureus* colonies were streaked on blood agar (BA) (Quad Five, Ryegate, MT) and TSB agar and incubated at 37°C for 24 h.

Following incubations on BA and TSB agar from the primary and secondary MSA plates, colonies were subjected to catalase (Sigma, St. Louis, MO) and coagulase testing (BactiStaph^®^ Latex 450, Remel Inc.). Positive tests were considered diagnostic for *S*. *aureus*. The identification of *S*. *aureus* was also confirmed genetically by PCR amplification and sequencing of a fragment of the *spa* gene for 1598/1662 (96%) of the isolates as done previously [38]. The second MSA plates streaked from the overnight liquid broth cultures were examined for additional growth, and colonies with *S*. *aureus* morphology were isolated and tested as above. Once isolates were defined as *S*. *aureus*, their respective susceptibilities to methicillin were determined using the E-test^®^ (AB Biodisk, Biomerieux, I’Etoile, France). Methicillin resistance was defined by growth at antibiotic concentrations ≥4 μg/ml. All confirmed *S*. *aureus* isolates were stored at -80°C [38].

### Definition of the *S*. *aureus* carriage phenotypes

Carriage status was determined for individuals from whom nasal swabs were collected at two time points, 2 weeks apart (14±3 days) as described previously [39]. Carriers were defined by *S*. *aureus* positive cultures at either visit, and intermittent carriers were *S*. *aureus* positive at either the first or second visit but not both. Non carriers were negative for *S*. *aureus* at both visits [39].

### Genome-wide association studies and generation of whole genome imputation data

Subjects (n = 858) were eligible for this study because of prior participation in genome-wide association studies for diabetes [40]. Genotyping was performed at the Center for Inherited Disease Research using the Affymetrix Genome-wide SNPArray 6.0 assay with sample- and SNP-level genotyping quality control performed as described in Below *et al*. [40]. Imputations were carried out in the full Starr County sample, cleaned of ethnic outliers and including 1,616 unrelated (pairwise identity by descent ≤ 0.3) [41] individuals of which 858 met inclusion criteria in the present study. A set of autosomal scaffold SNPs were selected to drive imputation by excluding those with: 1) minor allele frequency <1%, 2) Hardy-Weinberg p-values < 10^−4^ in the full sample 3) missingness >10% in the full sample and 4) all ambiguous strand (AT/CG) SNPs. Individual-level missingness is <5% in all samples. 603,042 scaffold SNPs were carried forward into a two-step imputation strategy: i) pre-phasing using the program SHAPEIT [42] and ii) Imputation from the reference panel into the estimated haplotypes with IMPUTE v2 [43–45]. Imputations were done in conjunction with the T2D-GENES consortium as part of a larger set of some 13,000 multiethnic samples. SNPs with imputation quality ≥ 0.8 and minor allele frequency > 0.05 were carried forward for single variant analyses. Population stratification was evaluated using EIGENSOFT on a subset of directly genotyped SNPs pruned for local and long distance linkage disequilibrium as described in Patterson *et al*. [46].

Analyses were conducted by comparing persistent *S*. *aureus* carriers to noncarriers or intermittent carriers to noncarriers. Persistent carriers were defined as unrelated [41] individuals passing genotyping quality control and testing positive for colonization of *S*. *aureus* at both of two time points, 11 to 17 days apart (n = 141). Genes located within 50 kilobases of signals comprised of at least 4 SNPs and study-based minor allele frequency > 0.05 with a *p* value <10^−5^ were considered suggestively significant. For each region showing association, we identified a sentinel marker, defined as the most significant SNP meeting all quality control thresholds (locus zoom plots, Figs A-L, in S1 File).

Associations of the imputed genetic markers with *S*. *aureus* carrier status were tested with the program SNPTEST v2 [44] using frequentist association tests, based on an additive model. To control for genotype uncertainty, we used the missing data likelihood score test (the *score* method). All association analyses corrected for ancestry using the first and second principal components from EIGENSOFT as covariates, and all analyses were run once including diabetes status as a covariate and once excluding diabetes status in the model.

### Generation and analysis of whole exome single variants

Whole exome sequence data were available for a subset of 792 participants (131 persistent carriers, 88 intermittent carriers, and 573 non-carriers, as defined above). These were part of a larger group sequenced as part of the T2D-GENES Consortium at the Broad Institute using Agilent Truseq capture reagents on Illumina HiSeq2000 instruments.

**Fig 1:**
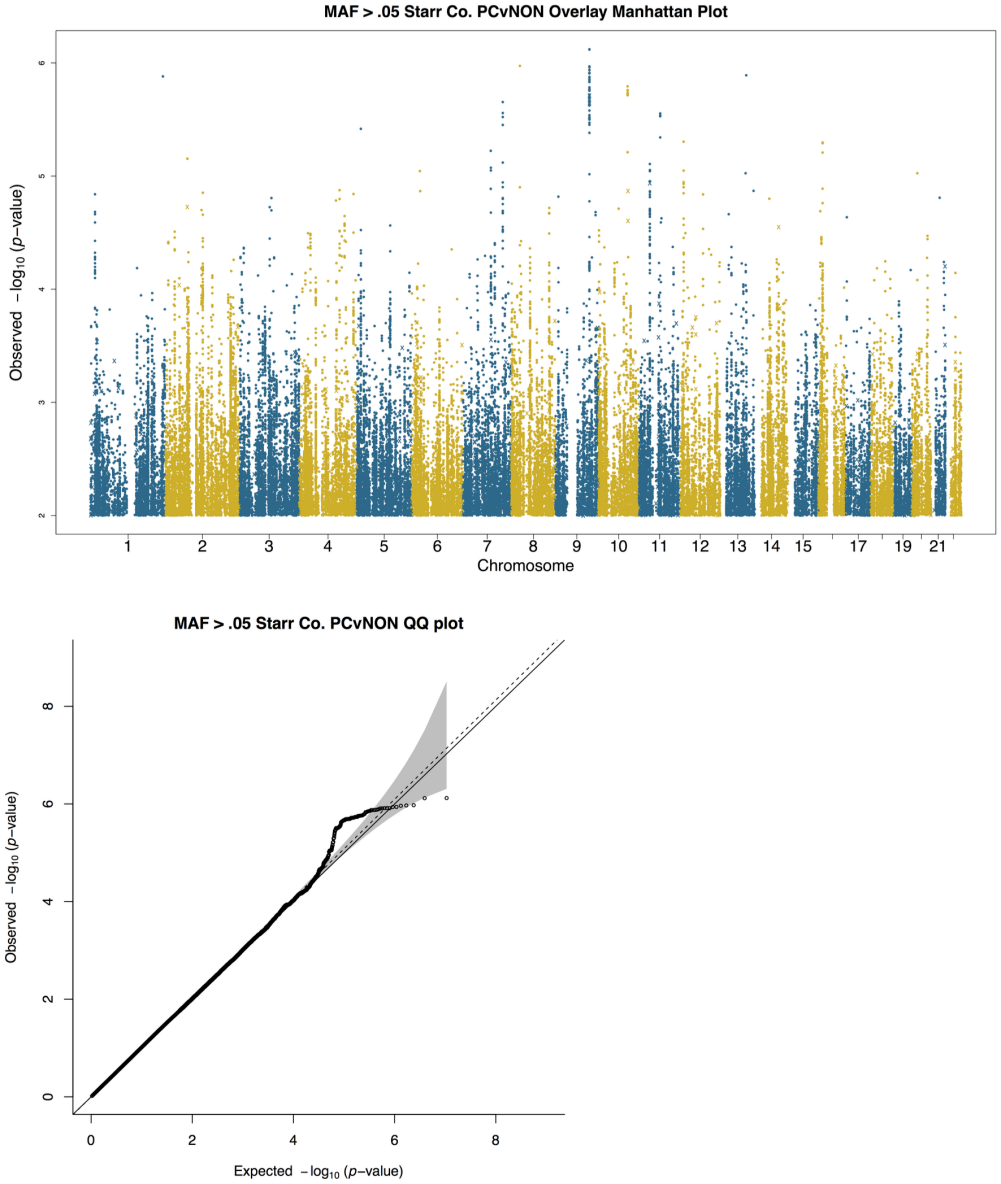
Manhattan (a) and QQ plots (b) of results of single variant logistic regression of persistent *S*. *aureus* carriage versus non-carrier, including PC1 and PC2 as covariates. The x-axis represents the chromosome number and each dot represents a single polymorphic variant with minor allele frequency greater than 0.05. QQ plot shows the observed versus expected p-values for the same variants shown in (a). Grey shading indicates the 95% confidence interval, the solid line indicates the expected null distribution, and the dotted line indicates the slope after lambda correction for genomic control. The 1,011 common variants identified by whole exome sequencing are shown as x’s in the Manhattan plots.

**Fig 2:**
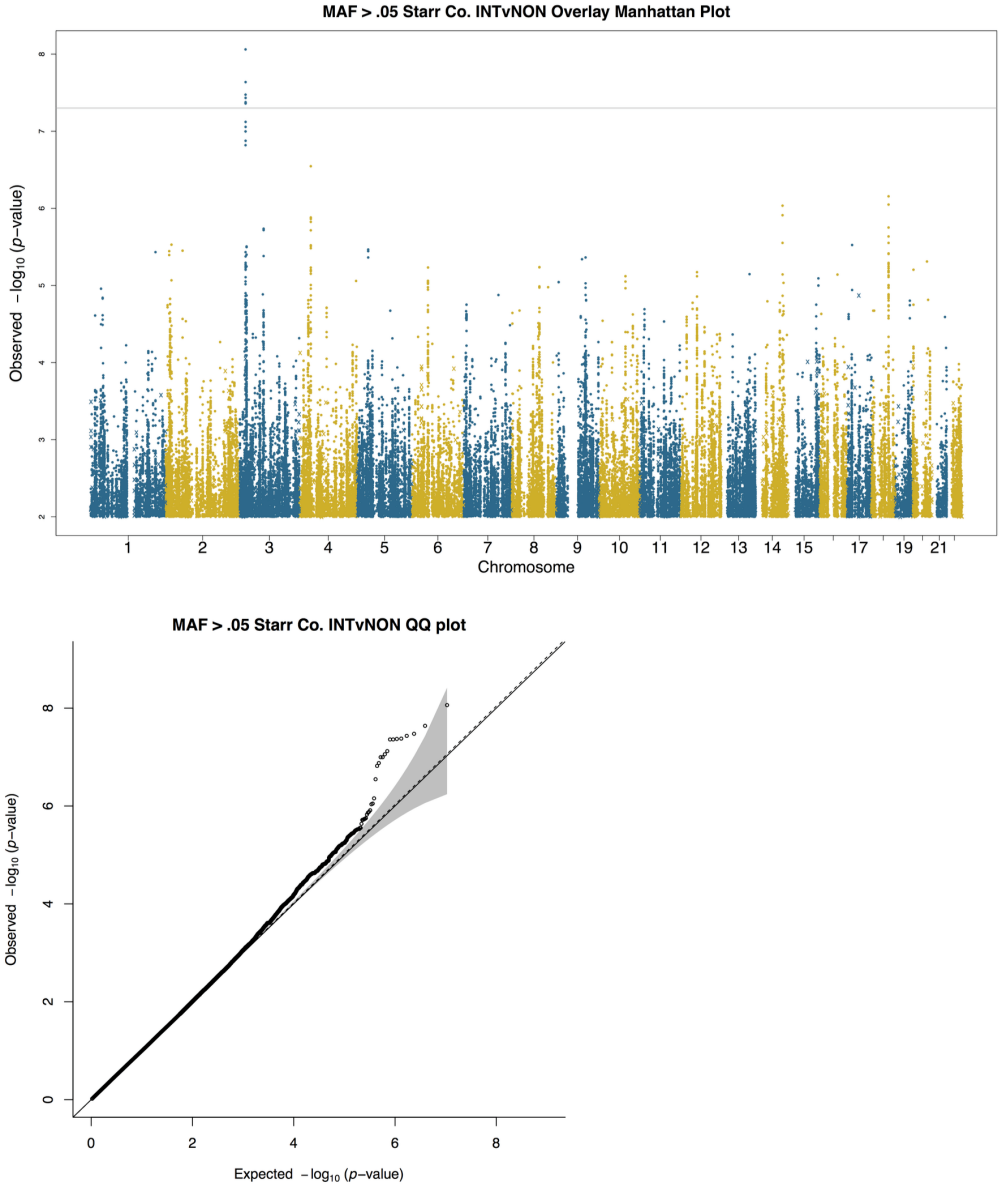
Manhattan (a) and QQ plots (b) of results of single variant logistic regression of intermittent *S*. *aureus* carriage versus non-carrier, including PC1 and PC2 as covariates. The x-axis represents the chromosome number and each dot represents a single polymorphic variant with minor allele frequency greater than 0.05. QQ plot shows the observed versus expected p-values for the same variants shown in (a). Grey shading indicates the 95% confidence interval, the solid line indicates the expected null distribution, and the dotted line indicates the slope after lambda correction for genomic control. The 1,011 common variants identified by whole exome sequencing are shown as x’s in the Manhattan plots.

Association tests of the 1,011 common (minor allele frequency > 0.05) single variants present in the exome sequence data were performed using logistic regression in the program PLINK v2 [47]. As above, association analyses were corrected for ancestry using the first and second principal components, and all analyses were run including and excluding diabetes status as a covariate in the model. These results were combined with the imputed data results in common Manhattan plots (Figs 1 and 2 and Figs M-N in S1 File).

### Gene-based analysis of whole exome sequence data

We used the Variant Annotation Analysis and Search Tool (VAAST) to identify genes associated with increased risk of *S*. *aureus* colonization [48, 49]. For quality-control purposes, we removed sites with missingness >10% in the full sample. We also used the rate option to set the maximum expected disease allele frequency to 0.05, as we expect to be powered to detect effects of common variants in single variant tests. The top two principle components from EIGENSOFT were used as covariates in all VAAST analyses, and analyses were performed with and without diabetes status as a covariate, as above. Statistical significance was assessed using a covariate-adjusted randomization test as previously described [50, 51]; p-value confidence intervals were calculated using a Poisson approximation based on the number of successes in the randomization test. Genome-wide significance thresholds for the gene-based tests were calculated from the number of genes tested (0.05/18665 = 2.68×10^−6^).

## Results

### Population demographics and *S*. *aureus* carriage determination

Carriage status was established by collecting and analyzing swabs for the presence of *S*. *aureus* on 2 occasions from a single nostril 11–17 days apart on 858 Mexican Americans from Starr County, TX, USA [39]. A summary of demographic information for these individuals, who were eligible due to prior participation in a genome-wide association study for type 2 diabetes, are presented in Table 1 [40]. Participants testing positive for *S*. *aureus* on 2 separate occasions were defined as persistent carriers (n = 141), participants testing positive once were defined as intermittent carriers (n = 97), and participants testing negative on both occasions were defined as non-carriers (n = 620) as previously described [38, 39].

**Table 1:** Demographic information for study participants by *S*. *aureus* carriage phenotype.

	Persistent Carrier	Intermittent Carrier	Non Carrier	Total
Total	141 (131)^A^	97 (88)	620 (573)	858 (792)
% Female	70.9	76.3	69.2	70.3
% Diabetes	52.5	62.9	48.7	50.9
Mean BMI	32.7 ±9.5	33.5 ±11.7	32.4 ±10.5	32.4 ±10.5
Mean Age	53.7 ±12.8	56.5 ±14.8	54.2 ±13.2	54.4 ±13.3
Mean hbA1C	6.8 ±2.4	7.2 ±2.7	6.6 ±2.3	6.7 ±2.4

^A^Number of individuals with available exome data.

### Single variant association tests

Single variant association tests of persistent *S*. *aureus* carriage identified 5 loci as suggestively significant (p value ≤ 10^−5^, as defined in the Methods) are summarized in Fig 1 and Table 2, namely *MKLN1* (muskelin 1), *SORBS1* (sorbin and SH3 domain containing 1), *SLC1A2* (solute carrier family 1) SORBS1, a region intergenic between *EPB41L4B* (erythrocyte membrane protein band 4.1 like 4B) and *PTPN3* (cytoskeletal-associated protein tyrosine phosphatase), and a region downstream of *FGF3* (fibroblast growth factor 3). *MKLN1* encodes an intracellular mediator of cell morphology and cytoskeletal responses [52, 53]. *SORBS1* is involved in insulin signaling and *SLC1A2* is a member of the solute transporter family. *EPB41L4B* and *PTPN3* are involved in membrane-cytoskeletal interactions while *FGF3* is a member of the fibroblast growth factor family of genes. *MKLN1* has been previously associated with childhood asthma [54], *SORBS1* with suicide risk (46) and childhood obesity in Hispanics [55], *SLC1A2* with fatty acid levels [56], essential tremor [57–59], and other traits [58, 59], *EPB41L4B* with wound healing [60], *PTPN3* with cancer [61], and *FGF3* with breast cancer [62] and deafness [63, 64]. Whole exome sequencing identified 1,011 common variants (minor allele frequency > 0.05). These are shown as x’s in the Manhattan plots. In no case did any of these variants reach a suggestive level indicating that it is unlikely that there are common protein-coding variants of substantial effect. LocusZoom [65] plots for each top locus highlight LD (linkage disequilibrium) patterns among the top SNPs and show multiple SNPs in LD blocks being associated (Figs A-E in S1 File).

**Table 2:** SNPs reaching suggestive significance in single variant logistic regression for persistent *S*. *aureus* carrier vs. non-carrier (top) and intermittent *S*. *aureus* carrier vs. non-carrier (bottom), including PC1 and PC2 as covariates.

					Persistent Carrier (PC) vs. Non Carrier Eigenscore 1,2	Intermittent Carrier (INT) vs. Non Carrier Eigenscore 1,2							
SNP	Chr	Position	Risk allele	Non-risk allele	OR (95% CI) [p value]	OR (95% CI) [p value]	IMPUTE2 info score	Freq (all)	Freq (PC)	Freq (INT)	Freq (controls)	Genes within 50 kb of locus	Location of sentinel SNP
rs118047622	7	130819082	C	G	**2.57 (1.74–3.80) [2.22E-06]**	1.72 (1.08–2.75) [2.25E-02]	0.89	0.16	0.24	0.19	0.13	*LINC-PINT*, *MKLN1*	Intronic *MKLN1* ^A^
rs138799235	9	112129775	C	T	**3.00 (1.94–4.63) [7.57E-07]**	1.04 (0.60–1.79) [8.91E-01]	0.98	0.11	0.19	0.09	0.09	*EPB41L4B* ^B^, *PTPN3* ^C^	Intergenic *EPB41L4B* and *PTPN3*
rs4918947	10	97293912	A	G	**3.91 (2.24–6.83) [1.61E-06]**	0.69 (0.32–1.48) [3.37E-01]	0.99	0.06	0.12	0.03	0.05	*SORBS1* ^D^, *ALDH18A1* ^E^	Intronic *SORBS1*
rs2421770	11	35320880	C	G	**1.83 (1.40–2.38) [7.81E-06]**	1.25 (0.92–1.72) [1.57E-01]	0.98	0.65	0.76	0.67	0.62	*SLC1A2* ^F^	Intronic *SLC1A2*
rs734102	11	69624482	C	T	**3.68 (2.13–6.35) [2.79E-06]**	1.51 (0.83–2.75) [1.80E-01]	0.99	0.94	1.00	0.95	0.92	*FGF3* ^G^, *FGF4* ^G^	Downstream *FGF3*
rs61440199	3	20111546	A	G	2.63 (1.36–5.11) [4.20E-03]	**8.68 (4.16–18.13) [8.68E-09]**	0.99	0.05	0.07	0.12	0.03	*KAT2B* ^H^, *SGOL1* ^I^, *PP2D1* ^J^, *SGOL1-AS1* ^K^, *MIR3135A* ^L^	Intronic *KAT2B*
rs7611684	3	23482812	A	G	1.31 (0.84–2.06) [2.32E-01]	**3.26 (1.98–5.35) [3.12E-06]**	0.96	0.11	0.12	0.21	0.10	*UBE2E2* ^M^	Intronic *UBE2E2*
rs11127662	3	79771987	G	C	1.27 (0.73–2.20) [3.99E-01]	**4.32 (2.37–7.87) [1.84E-06]**	0.99	0.07	0.07	0.15	0.06	*ROBO1* ^N^	Intronic *ROBO1*
rs16993852	4	37737989	T	C	3.48 (1.85–6.52) [1.03E-04]	**6.93 (3.31–14.52) [2.84E-07]**	0.98	0.06	0.09	0.12	0.04	*RELL1* ^O^	Intergenic *RELL1* and *PGM2* ^P^
rs222458	6	52890625	A	G	1.02 (0.53–1.94) [9.53E-01]	**4.77 (2.39–9.49) [8.71E-06]**	1.00	0.05	0.04	0.11	0.04	*GSTA4* ^Q^, *RN7SK* ^R^, *ICK* ^S^, *RN7SL244P* ^T^, *FBXO9* ^U^	Intronic *ICK*
rs1682522	14	87641837	T	A	1.05 (0.70–1.59) [7.99E-01]	**3.16 (1.99–4.99) [9.21E-07]**	0.91	0.14	0.13	0.25	0.12	*LOC283585* ^V^, *GALC* ^W^	Intergenic *LOC283585* and *GALC*
rs8088420	18	56511908	C	T	1.17 (0.85–1.60) [3.45E-01]	**2.48 (1.73–3.55 [6.98E-07])**	0.94	0.27	0.28	0.41	0.25	*RNU6-219P* ^X^, *ZNF532* ^Y^, *RN7SL112P* ^Z^	Intergenic *MALT1* ^AA^ and *ZNF532*

^A^Muskelin 1; encodes an intracellular mediator of cell morphology and cytoskeletal responses [52, 53].

^B^Erythrocyte membrane protein band 4.1 like 4B (also known as Ehm2); involved in membrane and cytoskeletal interactions [60].

^C^Cytoskeletal-associated protein tyrosine phosphatase; regulates cell growth, differentiation, mitotic cycle, and oncogenic transformation [61].

^D^Sorbin and SH3 domain containing 1; encodes a CBL-associated protein which functions in the signaling and stimulation of insulin [132].

^E^Aldehyde Dehydrogenase 18 Family, Member A1; Diseases associated with ALDH18A1 include cutis laxa, autosomal recessive, type iiia and aldh18a1-related cutis laxa [149–151].

^F^Solute carrier protein 1; associated with fatty acid levels, essential tremor and other traits [56–59].

^G^Fibroblast growth factors 3 and 4; cell growth and breast cancer [62–64].

^H^Lysine acetlytransferase 2B (also known as PCAF or p300/CBP associated factor); associated with various phenotypes including inflammatory responses to S. aureus [66–70].

^I^Shugoshin-Like 1 cancer; associated with the cell cycle [102].

^J^Protein Phosphatase 2C-Like Domain Containing 1; function unknown.

^K^SGOL1 Antisense RNA 1; RNA gene. Function undefined.

^L^MicroRNA 3135a; Function undefined.

^M^Ubiquitin-conjugating enzyme E2E; associated with gestational and type 2 diabetes [71–73].

^N^Roundabout, axon guidance receptor, homolog 1; encodes a member of the immunoglobulin gene superfamily and neuronal precursor cell migration [74–77].

^O^RELT-like 1; tumor necrosis receptor family member also involved in immune regulation [109].

^P^Phosphoglucomutase 2; Catalyzes the conversion of the nucleoside breakdown products ribose-1-phosphate and deoxyribose-1-phosphate [genecards.org].

^Q^Glutathione S-Transferase Alpha 4;involved in cellular defense against toxic, carcinogenic, and pharmacologically active electrophilic compounds [genecards.org].

^R^RNA, 7SK Small Nuclear; RNA gene [genecards.org].

^S^Intestinal cell [MAK-like] kinase; associated with the cell cycle [101, 104].

^T^RNA, 7SL, Cytoplasmic 244, Pseudogene [genecards.org].

^U^F-Box Protein 9, associated with adipocyte differentiation and innate immunity [108,111].

^V^RNA gene; non coding RNA [genecards.org].

^W^b-galactocerebroside; responsible for Krabbe disease [78, 79].

^X^Pseudogene [genecards.org]

^Y^Zinc finger protein 532; affects adipogenesis differentiation [110].

^Z^RNA; unknown function.

^AA^Mucosa associated lymphoid tissue lymphoma translocation gene 1; associated with immunodeficiency and multiple sclerosis [80–83]

In addition, we carried out single variant association tests of intermittent carriage of *S*. *aureus*, defined as individuals testing positive for *S*. *aureus* colonization at either visit compared to non *S*. *aureus* carriers (Fig 2, Table 2). The 7 regions suggestively associated (as defined above) with intermittent carriage include a genome-wide significant finding on chromosome 3 at rs61440199 (*p* value 8.68 x 10^−9^) that is intronic to *KAT2B* (lysine acetyltransferase 2B) (also known as *PCAF*; p300/CBP-associated factor), a gene associated with post-traumatic stress disorder [66], mean arterial blood pressure [67], adipogenesis [68], development of T regulatory cells [69], and recently shown to be a potential regulator of inflammatory responses following infection with *S*. *aureus* in a mouse model of disease (Table 2) [70]. Other signals were at or near *UBE2E2* (ubiquitin-conjugating enzyme E2E 2), a gene that has been associated with risk to gestational and type 2 diabetes [71–73], *ICK* (intestinal cell [MAK-like] kinase), and *ROBO1* (roundabout, axon guidance receptor, homolog 1), which encodes a member of the immunoglobulin gene superfamily and plays a role in axon guidance and neuronal precursor cell migration (Table 2). A SNP highly correlated with *ROBO1* expression in the brain has been reproducibly associated with reading disabilities [74, 75], and SNPs mapped to *ROBO1* have been associated with levels of liver enzymes [76] and other pQTLs [77]. Three sentinel SNPs were intergenic between (RELT-like 1) and *PGM2* (phosphoglucomutase 2), between genes *LOC283585* and *GALC*, and between *ZNF532* (zinc finger protein 532) and *MALT1* (mucosa associated lymphoid tissue lymphoma translocation gene 1) (Table 2). *GALC* encodes the enzyme β-galactocerebrosidase, mutations in which are responsible for Krabbe disease [78, 79]. Homozygous mutations in *MALT1* have been associated with immunodeficiency [80–82] (Table 2). *MALT1* has also been associated with multiple-sclerosis [83]. No common (minor allele frequency >0.05) variants in the whole exome sequencing data reached *p* value < 10^−5^ (shown as x’s in the Manhattan plot, Fig 2). LocusZoom plots for each top locus highlight LD patterns among top SNPs (Figs F-L in S1 File). It is notable that the signals for persistent carriage of *S*. *aureus* appear to be largely independent of signals for intermittent carriage of *S*. *aureus*. Of all top findings, only rs61440199 (*KAT2B*) and rs16993852 (*RELL1*) show nominal evidence of association in both persistent and intermittent carriage of *S*. *aureus*. Diabetes stratified and non-stratified analyses of both persistent and intermittent carriage gave highly concordant results across all analyses (Figs M-N in S1 File).

### Gene-based tests of functional variants

The program VAAST [49, 50] was used to identify genes enriched for functional rare variation in cases based on next generation whole exome sequence data. In the analysis of persistent carriers (131 cases, Table 1) versus non-carriers (573 controls, Table 1) of *S*. *aureus*, one gene, *FAM123C* (APC membrane recruitment protein 3), approached genome-wide significance (*p* value 6.50 x 10^−6^) (Table 3). Other top gene-based findings include *NGEF* (neuronal guanine nucleotide exchange factor, *p* value 1.22 x 10^−5^), *CCDC69* (coiled-coil domain containing 69, *p* value 1.40 x 10^−5^), *ERP29* (endoplasmic reticulum protein 29, *p* value 3.72 x 10^−5^), and *TSGA10IP* (testis-specific protein 10-interacting protein, *p* value 7.45 x 10^−5^ (Table 3 and Fig O in S1 File). In the analysis of intermittent carriers (88 cases, Table 1) versus non-carriers (573 controls, Table 1) top gene-based findings included *SLC4A4* (bicarbonate cotransporter, member 4, *p* value 2.27 x 10^−4^), *TSPAN11* (tetraspanin 11, *p* value 1.98 x 10^−4^), *TPO* (thyroid peroxidase, *p* value 4.05 x 10^−4^), *ZNF280D* (zinc finger protein 280D, *p* value 3.76 x 10^−4^), and *CSF2RB* (colony stimulating factor 2 receptor, beta, low-affinity, *p* value 4.15 x 10^−4^) (Table 3 and Fig P in S1 File). Specific variant enrichment and predicted function of variants driving top gene-based findings are shown in Table 3 and gene functions are discussed below.

**Table 3:** Top findings from gene-based burden tests of rare functional variation in VAAST for persistent *S*. *aureus* carriers versus non-carriers (top) and intermittent *S*. *aureus* carriers versus non-carriers (bottom); including PC1, and PC2 as covariates.

Gene ID	*p* value, Persistent Carrier (PC) vs. Non Carrier (Eigenscore 1,2)	*p* value, Intermittent Carrier (INT) vs. Non Carrier (Eigenscore 1,2)	Variant Location	Mutation	Count (PC)	Count (INT)	Count (control)	PC vs Non OR (95% CI)	INT vs Non OR (95% CI)	Mutation Taster Prediction
*FAM123C*	6.5x10^−6^ (4.93x10^−6^, 8.17x10^−6^)	0.156 (0.112, 0.205)	chr2:131520672	p.D343H	3/253	0/176	0/1124	-	-	polymorphism
			chr2:131520276–131520278	p.211_211del	2/258	0/174	0/1130	-	-	polymorphism
			chr2:131520231	p.R196W	0/256	1/173	0/1098	-	-	polymorphism
			chr2:131520255	P204A	2/256	0/170	0/1108	-	-	polymorphism
*NGEF*	1.22x10^−5^ (9.54x10^−6^, 1.5x10^−5^)	0.123 (0.084, 0.166)	chr2:233744262	p.M690I	3/259	1/175	0/1146	-	-	polymorphism
			chr2:233756151	p.D397N	2/258	0/176	0/1146	-	-	damaging
			chr2:233757708	p.V348M	1/261	0/176	0/1146	-	-	damaging
*CCDC69*	1.4x10^−5^ (1.12x10^−5^, 1.7x10^−5^)	0.0399 (0.0254, 0.0561)	chr5:150565006	p.R198W	3/259	0/176	0/1146	-	-	polymorphism
			chr5:150567017	p.L108P	12/240	6/166	13/1109	4.27 (1.92, 9.46)	3.08 (1.16, 8.22)	damaging^A^
*ERP29*	3.72x10^−5^ (2.79x10^−5^, 4.71x10^−5^)	0.0193 (0.0124, 0.027)	chr12:112460215	p.K182R	7/249	2/174	5/1129	6.35 (2.00, 20.16)	2.60 (0.50, 13.48)	polymorphism
			chr12:112460316	p.F216L	1/261	0/176	0/1146	-	-	damaging
			chr12:112459997	p.K109N	1/261	1/175	0/1146	-	-	damaging
			chr12:112460195	p.E175D	1/257	1/173	0/1132	-	-	damaging
*TSGA10IP*	7.45E-05 (5.59x10^−5^, 9.43x10^−5^)	1 (1, 1)	chr11:65714925	p.A210V	30/214	9/155	56/1008	2.52 (1.58, 4.03)	1.05 (0.51, 2.16)	NA
			chr11:65715005	p.R237S	1/257	0/176	0/1140	-	-	NA
*SLC4A4*	0.236 (0.182, 0.295)	2.27x10-4 (1.81x10-4, 2.76x10-4)	chr4:72205078	p.T38I	0/262	1/175	0/1146	-	-	damaging
			chr4:72215759	p.R130W	1/261	2/174	1/1145	4.39 (0.27, 70.37)	13.16 (1.19, 145.92)	damaging
			chr4:72316967	p.G380D	1/257	4/172	4/1140	1.11 (0.12, 9.96)	6.63 (1.64, 26.75)	damaging
			chr4:72319250	p.A410V	0/262	1/175	0/1146	-	-	damaging
			chr4:72363275	p.G634R	0/260	1/171	0/1136	-	-	damaging
*TSPAN11*	0.113 (0.0757, 0.154)	1.98x10-4 (1.55x10-4, 2.43x10-4)	chr12:31132507	p.D120N	0/262	1/175	0/1146	-	-	damaging
			chr12:31135497	p.D163N	1/257	4/172	1/1145	4.46 (0.28, 71.47)	26.63 (2.96, 239.65)	damaging
*TPO*	1 (1, 1)	4.05x10-4 (3.19x10-4, 4.97x10-4)	chr2:1488616	p.L356F	1/261	2/174	6/1140	0.73 (0.09, 6.07)	2.18 (0.44, 10.91)	damaging
			chr2:1497657	p.V445M	0/256	7/169	5/1135	-	9.40 (2.95, 29.96)	polymorphism
			chr2:1499870	p.M533V	0/250	7/161	4/1110	-	12.07 (3.49, 41.67)	polymorphism
			chr2:1544436	p.G853R	1/261	2/174	4/1142	1.09 (0.12, 9.83)	3.28 (0.60, 18.05)	polymorphism
*ZNF280D*	0.0342 (0.0208, 0.0493)	3.76x10-4 (2.93x10-4, 4.65x10-4)	chr15:56923895	p.G901V	1/261	3/173	13/1133	0.33 (0.04, 2.56)	1.51 (0.43, 5.36)	damaging
			chr15:56974513	p.Q302K	0/254	1/171	0/1116	-	-	polymorphism
			chr15:56981270	p.N237I	0/260	2/174	0/1144	-	-	damaging
			chr15:56981286	p.C232R	0/260	1/175	0/1144	-	-	damaging
			chr15:56923952	p.Q882R	1/259	0/176	0/1146	-	-	damaging
			chr15:56924054	p.I848T	1/261	0/176	0/1146	-	-	polymorphism
			chr15:56958707	p.I614T	1/261	0/176	0/1146	-	-	damaging
			chr15:56993196	p.I93V	1/259	0/174	0/1142	-	-	damaging
*CSF2RB*	7.04x10^−4^ (4,77x10^−4^, 9.53xx10^−4^)	4.15x10-4 (3.27x10-4, 5.08x10-4)	chr22:37326443	p.E249Q	14/246	21/153	65/1077	0.94 (0.52, 1.71)	2.27 (1.35, 3.83)	polymorphism
			chr22:37326794	p.D312N	4/258	3/173	2/1144	8.87 (1.62, 48.68)	9.92 (1.65, 59.79)	polymorphism
			chr22:37328885	p.R364L	1/255	1/173	0/1128	-	-	polymorphism
			chr22:37331407	p.V444M	0/262	1/175	0/1144	-	-	polymorphism
			chr22:37319324	p.Y39H	1/261	0/176	0/1144	-	-	damaging
			chr22:37326794	p.D312N	4/258	3/173	2/1144	8.87 (1.62, 48.68)	9.92 (1.65, 59.79)	polymorphism
			chr22:37328885	p.R364L	1/255	1/173	0/1128	-	-	polymorphism
			chr22:37329979	p.G420S	2/254	0/176	1/1141	8.98 (0.81, 99.47)	-	polymorphism
			chr22:37334510	p.P887R	1/257	0/176	0/1140	-	-	polymorphism

^A^Predicted to be a disease causing polymorphism by CASM (Conservation-Controlled Amino Acid Substitution Matrix Prediction)(72).

As in the single variant analysis of 1000 Genomes imputed data and common variants from the exome sequence data, the gene-based findings in analyses of intermittent carriers of *S*. *aureus* appear to be largely independent between analyses of persistent versus intermittent carriage groups (Table 3). Only genes *CCDC69* and *ZNF280D* reach nominal levels of significance (*p* value < 0.05) in both tests. *CSF2RB* shows suggestive enrichment of missense variation in both analyses, and may constitute a gene involved in general *S*. *aureus* carriage susceptibility (Table 3). Shared signals should be interpreted with caution given that the non-carriage control group is the same in both tests, and thus the two tests are not strictly independent. As observed for the single variant association tests, diabetes stratified and non-stratified analyses gave highly concordant results across all analyses (Figs Q-R in S1 File). Manhattan and QQ plots suggest the type 1 error for both single variant and gene-based tests are well controlled (Figs M-R in S1 File).

We used the Disease Association Protein-Protein Link Evaluator (DAPPLE) [84] to identify interactions between proteins encoded by the top 5 candidate genes in the persistent versus non-carrier and intermittent versus non-carrier VAAST runs. DAPPLE searches for protein-protein interactions among a candidate gene list; a significant number of protein-protein interaction may indicate a shared molecular pathway relevant to *S*. *aureus* susceptibility. In the analysis of persistent carriers versus non-carriers we did not detect any direct protein-protein interactions. However, among the top 5 genes identified from the intermittent carriers versus non-carriers run, we found that *TPO* is directly interacting with *CSF2RB* (Fig S in S1 File). The p-value for observing at least one interaction among the top 5 genes is 0.008; the p-values for observing at least one interacting protein for *TPO* and *CSF2RB* are 0.015 and 0.014, respectively.

### Replication of previously identified loci

We compared our persistent carriage single variant and gene-based test results to all sites previously reported in genetic analyses of *S*. *aureus* (S1 Table) [20–23, 27, 30–32, 34, 35, 85]. When the variant in question was not present in our post-quality control imputed or exome sequenced variant lists, and therefore not analyzed in this study, we identified the best proxy variant by assessing linkage disequilibrium patterns in the Mexican-American (MEX) reference population within the 1000 Genomes Project data (release 27). In these cases, statistics for the variant with the highest linkage disequilibrium r^2^ are provided in S1 Table.

With the exception of *CDK7* (discussed below) our findings do not replicate the genes and variants described in 2 previously conducted genome wide association studies, possibly because of several differences between these prior studies and the current study (described in the Discussion)[34, 35]. We found suggestive evidence of association at rs4918120 (*p* value 0.034) a SNP previously identified by Nelson *et al*. [34] in Caucasian inpatients; however, we observed the opposite direction of effect of the T allele (odds ratio 0.70 versus 1.68, see S1 Table). Interrogation of our single variant test results for intermittent carriage at previously reported loci yielded replication at three loci identified by Ye *et al*. [35]: rs12696090 (*p* value 0.0214), rs7643377 (*p* value 0.0081), and rs9867210 (*p* value 0.0079), however as before; we find opposite direction of effect at each of these loci (S1 Table).

We also examined our gene-based test results for replication of previous findings at genes near previously associated SNPs and genes. *CDK7* (cyclin-dependent kinase) (gene-based *p* value 0.040) replicates findings by Ye *et al*. [35] who studied genetic risk of hospital-based *S*. *aureus* infection in Caucasians and identified *CDK7* using gene-based tests in the program VEGAS (S1 Table).

## Discussion

This was the first genome-wide association study of *S*. *aureus* carriage states in a community-based representative population. This approach is significantly different from previously described genome-wide association studies that were carried out in the context of *S*. *aureus* infections [34, 35]. We found genome-wide significance at 1 gene region and 11 other regions meeting suggestive levels of significance for association with persistent and intermittent carriage states by single variant analysis. We also reported the 5 top findings from gene-based tests of persistent and intermittent carriage. The lack of overlap in signals between gene-based tests of rare functional variation and single-variant tests suggested that genome-wide association signals were not driven by coding sequence variation. Non-genic regulatory factors affecting gene expression levels or post-translational modifications may also affect carriage phenotypes.

We found that top signals associated with persistent and intermittent carriage captured genes of different cellular functions. Genome-wide single variant analysis identified 5 gene regions suggestively associated with persistent carriage. Gene-based rare variant analysis identified 5 genes in association with persistent carriage. Near genome-wide significance was observed only for *FAM123C* (*p* value < 6.50 x 10^−6^). Each of these genes (except for *TSGA10IP*, which has not been previously described to our knowledge) was involved with cellular growth, tissue homeostasis, and/or cancer [86–92]. It should be noted however, that TSGA10IP (TSGA10 interacting protein) interacts with TSGA10, a protein also associated with cancer and that binds cytoskeletal proteins (*e*.*g*., vimentin and actin-γ1) [93, 94].

In analyses of persistent *S*. *aureus* carriage, all of the top 5 findings from gene-based tests and all regions identified in the single variant analysis harbored at least 1 gene associated with either regulation of cell growth or maintenance of cellular integrity (*e*.*g*., tight junctions) [95, 96]. Conversely, a minority of genes identified in previous genome-wide association studies of *S*. *aureus* infection were involved in cell cycle, cellular growth, or cellular integrity (S1 Table) [34, 35]. These differences are important for 2 reasons: i) carriage and infection are not mutually exclusive *i*.*e*., the *S*. *aureus* carriage status of individuals was not established in relation to the infections described in the previous genome-wide association studies, and ii) susceptibility to infections in hospital environments may not accurately reflect an individual's susceptibility to an infectious agent. Hospital environments in and of themselves place patients at increased risk for infections with numerous pathogens including *S*. *aureus*, an agent responsible for more healthcare-associated infections and surgical site infections than any other pathogen [97].

Genome-wide association analysis of intermittent carriage identified a different set of genes from those identified in association with persistent carriage. This analysis identified 7 gene regions. The top signal (rs61440199) was genome-wide significant (*p* value 8.68 x 10^−9^) and intronic to *KAT2B*. This gene was of particular interest since its expression in mice was affected by the nature of the infecting *S*. *aureus* strain [70]. In addition, *KAT2B* has been linked to immune function, cancer progression, and adipogenesis [68, 98–100]. The association of *KAT2B* with cancer progression/cell cycle was also shared by *SGOL*, *ROBO1*, and *ICK*, and represents the only functional overlap with genes identified with persistent and intermittent carriage of *S*. *aureus* [101–107]. The other themes observed in the context of genes associated with intermittent carriage were genes associated with both adipogenesis and inflammation/immunity (*KAT2B*, *ZNF532*, *RELL1*, *FOXO9*, *MALT1*) [68, 80, 98, 100, 101, 103, 108–112]. In light of sample ascertainment for diabetes in this cohort [40], a gene in 1 region, *UBE2E2*, was of interest because of prior associations with diabetes risk [71–73], however, stratification for diabetes provided highly concordant results with the unstratified analysis (data not shown). Our gene-based analyses did not model complications that present in diabetic patients (*e*.*g*., obesity, immune function, elevated blood glucose levels) that may alter susceptibility to intermittent carriage. The number of adipogenesis genes linked to intermittent carriage may be of significance in light of recent studies that identified a protective role for adipose tissue in a murine model of *S*. *aureus* skin infection, suggesting that immune factors produced by adipose tissues (*e*.*g*., antimicrobial peptides) may play a role in intermittent carriage [112].

Although gene-based analyses of rare functional variants failed to identify any genome-wide significant differences in association with intermittent carriage, a top signal, *CSF2RB*, demonstrated concordance of burden in both persistent and intermittent carriers (*p* value 7.04 x 10^−4^ and *p* value < 4.15 x 10^−4^, respectively). *CSF2RB* codes for CD131, the common β receptor subunit for IL-3, IL-5, and GM-CSF (granulocyte/monocyte colony stimulating factor) that in mice was shown to play a role in regulating Th2 type immune responses [113]. In addition, CD131 stimulated the recruitment of neutrophils (which are a key innate immune component) and controlled the homeostasis of tissue dendritic cells [114, 115]. In addition, DAPPLE analysis identified a significant protein-protein interaction between the *CSF2RB* and *TPO* gene products. *TPO* is critical to the production of thyroid hormones that can impact immune function and is also associated with mucinosis (myxedema), a disease characterized by increased glycosaminoglycan deposition in the skin [116, 117]. Other than *CSF2RB*, no other top finding in the gene-based tests were even modestly associated with both persistent and intermittent carriage.

Results from the 2 previously described genome-wide association studies identified a number of loci with statistical significance. However, those associations were for the most part not replicated in our studies or previous work [9, 18–23, 27, 30, 32, 34, 35, 85, 118]. Lack of replication between studies may be due to population differences, the impact of the respective colonizing/infecting *S*. *aureus* strains (and their relationship with distinct human genetic determinants), study design (*i*.*e*., *S*. *aureus* infection versus carriage), and the size of respective populations examined [9, 22]. Replication of 1 gene identified by gene-based tests was observed in the context of persistent carriage that identified *CDK7* (*p* value 0.041) from the VEGAS gene test conducted by Ye *et al*. (S1 Table). We also assessed gene-based evidence of replication in our analyses of intermittent carriage versus non-carriage and found no support for previously identified genes (data not shown).

Previous colonization studies have suggested that the 3 described staphylococcal carriage phenotypes (persistent, intermittent, and non-carriers) be modified to include only 2 carriage phenotypes: persistent carriers and intermittent/non-carriers [17]. However gene targets identified in the present *S*. *aureus* carriage genome-wide association study suggested that each phenotype is distinct. That the genome-wide association and rare variant analyses identified relatively little functional similarity between persistent and intermittent carriers may suggest underlying differences between these 2 carriage states. An alternate explanation is that these studies lacked sufficient power to identify common factors across the carriage states. Despite the recommendation of previous studies to consider intermittent and non-carriers as a single group, this reclassification would require ignoring the differences that exist between these 2 carriage states. It is clear, however, that persistent carriers represent the most distinct carriage state. This is supported by colonization studies that demonstrated that non-carriers (and decolonized intermittent carriers) artificially inoculated with *S*. *aureus* in the nares cleared the bacteria over a similar time period (4 days for non-carriers and 14 days for intermittent carriers) compared to persistent carriers (decolonized and then re-inoculated) that still harbored the *S*. *aureus* inoculum >154 days later [17]. Persistent carriers also had a different antibody profile against some staphylococcal virulence factors compared to the indistinguishable profile described for non-carriers and intermittent carriers [17]. In addition, persistent carriers that were decolonized and re-inoculated with a heterogeneous mix of *S*. *aureus* isolates were more likely to be re-colonized with their original colonizing isolate suggestive of an intimate association between the colonizing strain and the host [17].

This difference between persistent carriers and intermittent carriers (and intermittent carriers and non-carriers) is further accentuated by the function of the genes associated with the respective carriage states. Almost all determinants associated with persistent carriage were associated with cellular integrity, morphology, and growth, functions that directly hold the potential of impacting the host/pathogen interface that establishes environments permissive to persistent carriage.

Attachment to host surfaces is requisite for colonization and infection of host tissues by pathogens. *S*. *aureus* possesses an arsenal of adhesins capable of binding an array of host extracellular matrix (ECM) components. These components include fibrinogen, fibronectin, collagen, cytokeratin 10, elastin, heparan sulfate proteoglycans, von Willebrand factor, bone sialoprotein, vitronectin, and prothrombin that all facilitate the colonization of diverse tissues and accounts in part for the myriad of diseases than can result following infection with this pathogen [119–121]. It is not surprising therefore that host polymorphisms potentially affecting cellular integrity, morphology and growth could also impact colonization with different pathogens or strains of the same pathogen.

That various potential genes identified by the genome-wide association study (*e*.*g*., *ALDH18A1*, *EPB41L4B*, *FGF3*, and *FGF4*) and all but 1 gene identified in the rare variant analysis have been shown to possess tumorogenic potential should not be surprising since various genes shown to play roles in the progression of various cancers also play critical roles in wound healing, cellular migration, cellular integrity, and angiogenesis [60, 122, 123]. Polymorphisms in these gene products or any gene products with the potential of altering the structural integrity of the host cell could potentially impact staphylococcal colonization.

Focal adhesions represent large, multi-protein complexes that are closely associated with cell surface integrins that span the eukaryotic plasma membrane linking the cellular cytoskeleton to the ECM (surrounding the cell) [124]. Most integrins and their respective focal adhesions are expressed in the epidermis and regulate epithelial cell homeostasis by mediating cell adhesion processes (and signaling) critical to tissue repair following injury [124]. Of the gene targets identified in association with S. *aureus* persistent carriage, *EHM2*, *PTPN3*, *SORS1*, and *MKLN1* can impact the integrity of focal adhesions that in turn alters the cytoskeleton [53, 120, 124–133].

*SORBS1* encodes CAP (Cbl-Associated Protein) [129, 132, 134] that affects insulin receptor signaling and also functions as a cytoskeletal regulatory protein [129]. In fibroblasts, when CAP associates with actin stress fibers, focal adhesion kinase binds CAP, and CAP over-expression induces the development of actin stress fibers and focal adhesions that physically link intracellular actin bundles to the extracellular substrates of many cell types [127, 135]. Various pathogens like *S*. *aureus* usurp focal adhesions as a means of triggering their uptake by various non-professional antigen presenting cells, including epithelia/endothelial cells, osteoclasts, kidney cells, fibroblasts and keratinocytes [120]. *S*. *aureus* possess various fibronectin binding proteins (*e*.*g*., FnbpA, FnbpB, ClfA, ClfB) that facilitate coating the bacterial surface with this matrix molecule that in turn binds to α5β1 integrins resulting in the formation of a molecular bridge linking *S*. *aureus* to the host cell [125]. This interaction triggers the recruitment of focal adhesion proteins that further alter the cytoskeleton facilitating attachment, invasion, and the ability to persist in their hosts [125]. The importance of this interaction for the successful attachment/invasion of human cells by staphylococci was demonstrated by generating *fnbpA/fnbpB*-deficient *S*. *aureus* that less effectively infected epithelial cells and in a mastitis model caused less severe disease [136, 137]. Furthermore, cells unable to form focal adhesions were resistant to integrin α5β1-mediated cellular invasion by *S*. *aureus* [120, 127].

EHM2 is a member of the 4.1R, ezrin, radixin, moesin (FERM) protein superfamily consisting of over 40 proteins that contain the characteristic 3-lobed FERM domain on the N-terminus that binds various cell membrane-associated proteins and lipids and the spectrin/actin binding domain (SABD) at the C-terminus [126]. The *PTPN3* gene product also belongs to the FERM family and is a protein phospatase that is a structural constituent of the cytoskeletal shown to play a role in T cell activation, maintenance of tight junction integrity (between the cell membrane and the cytoskeleton) and both *EHM2* and *PTPN3* gene products are associated with focal adhesions [95, 128, 130, 133, 138]. *EHM2* expression has been observed on wounds undergoing healing (primarily at the wound's leading edge) functioning as a positive regulator of keratinocyte adhesion and motility in addition to affecting the rates of cellular invasion and adhesion to colla*gen* via regulation of matrix metaloprotease 9 (*MMP9*) *i*.*e*., *EHM2* knockdown cells expressed significantly reduced levels of MMP9. This is of interest in the context of *S*. *aureus* since up- or down-regulation of MMP9 levels has been shown to affect disease progression resulting from *S*. *aureus* infections, that is, MMP9 levels that are either too high or too low can negatively affect wound healing and *MMP9*-deficient mice poorly controlled *S*. *aureus* infections [60, 126, 131, 139–143]. In addition, MMPs play critical roles in tissue remodeling (including the maintenance of the ECM), altering immune cell migration and infiltration patterns, and impacted inflammation by exerting effects on cytokines and chemokines [143, 144]. As it relates to *S*. *aureus* colonization, a role for MMP9 has yet to be described; however, staphylococcal lipoteichoic acid has been shown to increase production of MMP9 in middle ear epithelial cells suggesting that increased MMP9 levels could be involved in progression of otitis media [141].

Unlike *EHM2*, *PTPN3*, and *SORS1*; the *MKLN1* gene product muskelin mediates ECM binding via complex mechanisms involving interactions between different thrombospondin-1 (TSP-1) domains and various ligands (expressed by different cell types) including integrins, proteoglycans, or integrin-associated proteins. Alterations to muskelin expression levels altered attachment to TSP-1 in association with subtle changes to the organization of focal contacts [53]. Since TSP-1 has also been shown to serve as a ligand for *S*. *aureus*, polymorphisms in *MKLN1* could alter staphylococcal binding or prevent clearance of *S*. *aureus* since TSP-1 breakdown products function as antimicrobial peptides (AMPs) that have broad antibacterial properties affecting both Gram-positive and -negative bacteria [145–148].

Homozygous mutations in *ALDH18A1* (or other genes *e*.*g*., *PYCR1*, *ATP6V0A2*) can result in a heteogenous group of rare diseases characterized by loose or wrinkly skin known as cutis laxa [149–151]. Histologic analysis of skin from cutis laxa patients identified reduced elastin levels with less-well defined collagen fibers lacking the characteristic wavy morphology. In addition, collagen I and III levels were significantly reduced, and fibroblasts harvested from the dermis presented with reduced growth rates [149]. The majority of studies that have examined genes resulting in this rare condition have only described case reports of patients with homozygous mutations, making it difficult to interpret how polymorphisms with a less pronounced phenotype present at the cytoskeletal level.

Although adherence to host surfaces also represents a component of intermittent carriage (*i*.*e*., the organism has to attach to host tissues even if this association is transient), the intermittent periods of carriage, carriage of different strains over time, carriage of multiple strains, and the reduced *S*. *aureus* inocula recoverable from the nares of intermittent carriers suggests that different determinants are associated with this phenotype [17]. This is emphasized by the observation that the majority of gene targets associated with intermittent carriage were also associated with immune function/inflammation.

Our data suggested that determinants associated with persistent carriage and intermittent carriage differed. A limitation to the present study was the analysis of only two nasal swabs to establish carriage. Even though Nouwen *et al*. established that the 'two-culture' rule was 93.6% reliable [39] and numerous studies have used this approach to establish *S*. *aureus* carriage phenotypes [20, 38, 118, 152–156] there exists room for classification error. Second, because only one nostril was sampled some participants may have been misclassified as intermittent or non carriers based on one study that described differences in *S*. *aureus* carriage between colonization [157]; however, two other studies did not identify any differences [158, 159]. It should be noted that samples that were collected and analyzed for the present study were of the ciliated pseudostratified columnar epithelium associate the inferior and middle concha and not the nonkeratinized, squamous epithelium present in the anterior nares and used to establish *S*. *aureus* carriage by other studies. Furthermore, due to population differences and power we should be cautious in making assumptions with regard to specific genes associated with respective carriage states. We should therefore further dissect the observation that persistent carriage of *S*. *aureus* is affected primarily by polymorphisms at the host/pathogen interface and that intermittent carriage is more likely impacted by environmental factors combined with the heterogeneity of the host immune response.

## Supporting Information

S1 File **Figs A-E. LocusZoom plots of each top finding in the single variant association analyses of persistent *S*. *aureus* carriage versus non-carrier. (A)** *EPB41L4B*, **(B)** *LINC-PINT*, **(C)** *SORBS1*, *ALDH18A1*, **(D)** *SLC1A2*, and **(E)** *FGF4*, *FGF3*. **Figs F-L. LocusZoom plots of each top finding in the single variant association analyses of persistent *S*. *aureus* carriage versus non-carrier. (F)** *KAT2B*, **(G)** *UBE2E2*, *MIR548AC*, **(H)** *ROBO1*, **(I)** *RELL1*, **(J)** *GSTA4*, *ICK*, *FBXO9*, **(K)** *LOC283585*, *GALC*, and **(L)** *ZNF532*. **Fig M**. **Manhattan (a) and QQ plots (b) of results of single variant logistic regression of persistent *S*. *aureus* carriage versus non-carrier, including diabetes, PC1, and PC2 as covariates.** The x-axis represents the chromosome number and each dot represents a single polymorphic variant with minor allele frequency greater than 0.05. QQ plot shows the observed versus expected p-values for the same variants shown in (a). Grey shading indicates the 95% confidence interval, the solid line indicates the expected null distribution, and the dotted line indicates the slope after lambda correction for genomic control. The 1,011 common variants identified by whole exome sequencing are shown as x’s in the Manhattan plots. **Fig N**. **Manhattan (a) and QQ plots (b) of results of single variant logistic regression of intermittent *S*. *aureus* carriage versus non-carrier, including diabetes, PC1, and PC2 as covariates.** The x-axis represents the chromosome number and each dot represents a single polymorphic variant with minor allele frequency greater than 0.05. QQ plot shows the observed versus expected p-values for the same variants shown in (a). Grey shading indicates the 95% confidence interval, the solid line indicates the expected null distribution, and the dotted line indicates the slope after lambda correction for genomic control. The 1,011 common variants identified by whole exome sequencing are shown as x’s in the Manhattan plots. **Figs O**. **Manhattan (a) and QQ plots (b) of results of gene-based burden tests of rare functional variation in VAAST for persistent *S*. *aureus* carriage versus non-carrier including PC1, and PC2 as covariates.** The x-axis represents the chromosome number and each dot represent one protein-coding gene. QQ plot shows the observed versus expected p-values for all protein-coding genes, grey shading represents 95% confidence interval, the red line indicates the null distribution of p-values. **Fig P**. **Manhattan (a) and QQ plots (b) of results of gene-based burden tests of rare functional variation in VAAST for intermittent *S*. *aureus* carriage versus non-carrier including PC1, and PC2 as covariates.** The x-axis represents the chromosome number and each dot represent one protein-coding gene. QQ plot shows the observed versus expected p-values for all protein-coding genes, grey shading represents 95% confidence interval, the red line indicates the null distribution of p-values. **Fig Q**. **Manhattan (a) and QQ plots (b) of results of gene-based burden tests of rare functional variation in VAAST for persistent *S*. *aureus* carriage versus non-carrier including diabetes, PC1, and PC2 as covariates.** The x-axis represents the chromosome number and each dot represent one protein-coding gene. QQ plot shows the observed versus expected p-values for all protein-coding genes, grey shading represents 95% confidence interval, the red line indicates the null distribution of p-values. **Fig R**. **Manhattan (a) and QQ plots (b) of results of gene-based burden tests of rare functional variation in VAAST for intermittent *S*. *aureus* carriage versus non-carrier including diabetes, PC1, and PC2 as covariates.** The x-axis represents the chromosome number and each dot represent one protein-coding gene. QQ plot shows the observed versus expected p-values for all protein-coding genes, grey shading represents 95% confidence interval, the red line indicates the null distribution of p-values. **Fig S**. **Protein-protein interactions among top-5 candidate genes in the gene-based test of intermittent carriersversus non-carriers analysis.** Red: genes that encode proteins with direct interactions to another top-5 candidate; blue: genes that encode proteins with second-degree interactions to another top-5 candidate; grey: genes that are not top-5 candidates, but encode proteins interacting with at least two top-5 candidates. The figure was generated using DAPPLE software.
(DOCX)

S1 Table Previous genes and SNPs associated with S. aureus carriage or infection. (XLSX)
