# Supplementary material for: Genome-Wide Association Study of Staphylococcus aureus Carriage in a Community-Based Sample of Mexican-Americans in Starr County, Texas
Source: PLoS One. 2015 Nov 16;10(11):e0142130. doi: 10.1371/journal.pone.0142130 (PMC4646511; doi:10.1371/journal.pone.0142130)
Supplement: S1 File — Figs A-E. LocusZoom plots of each top finding in the single variant association analyses of persistent S. aureus carriage versus non-carrier. (A) EPB41L4B, (B) LINC-PINT, (C) SORBS1, ALDH18A1, (D) SLC1A2, and (E) FGF4, FGF3. Figs F-L. LocusZoom plots of each top finding in the single variant association analyses of persistent S. aureus carriage versus non-carrier. (F) KAT2B, (G) UBE2E2, MIR548AC, (H) ROBO1, (I) RELL1, (J) GSTA4, ICK, FBXO9, (K) LOC283585, GALC, and (L) ZNF532. Fig M. Manhattan (a) and QQ plots (b) of results of single variant logistic regression of persistent S. aureus carriage versus non-carrier, including diabetes, PC1, and PC2 as covariates. The x-axis represents the chromosome number and each dot represents a single polymorphic variant with minor allele frequency greater than 0.05. QQ plot shows the observed versus expected p-values for the same variants shown in (a). Grey shading indicates the 95% confidence interval, the solid line indicates the expected null distribution, and the dotted line indicates the slope after lambda correction for genomic control. The 1,011 common variants identified by whole exome sequencing are shown as x’s in the Manhattan plots. Fig N. Manhattan (a) and QQ plots (b) of results of single variant logistic regression of intermittent S. aureus carriage versus non-carrier, including diabetes, PC1, and PC2 as covariates. The x-axis represents the chromosome number and each dot represents a single polymorphic variant with minor allele frequency greater than 0.05. QQ plot shows the observed versus expected p-values for the same variants shown in (a). Grey shading indicates the 95% confidence interval, the solid line indicates the expected null distribution, and the dotted line indicates the slope after lambda correction for genomic control. The 1,011 common variants identified by whole exome sequencing are shown as x’s in the Manhattan plots. Figs O. Manhattan (a) and QQ plots (b) of results of gene-based burden tests of [file pone.0142130.s001.docx]

**S1 File**

**Fig A**

**
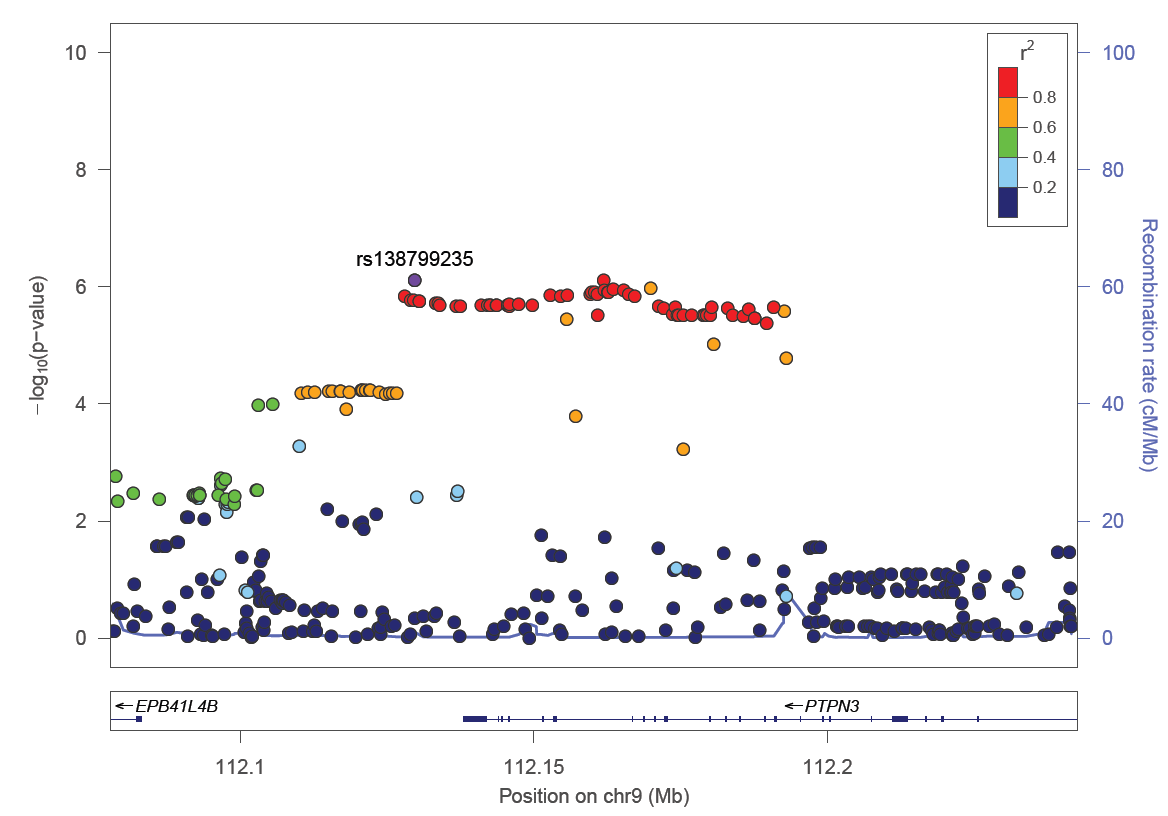
**

**Fig B**

**
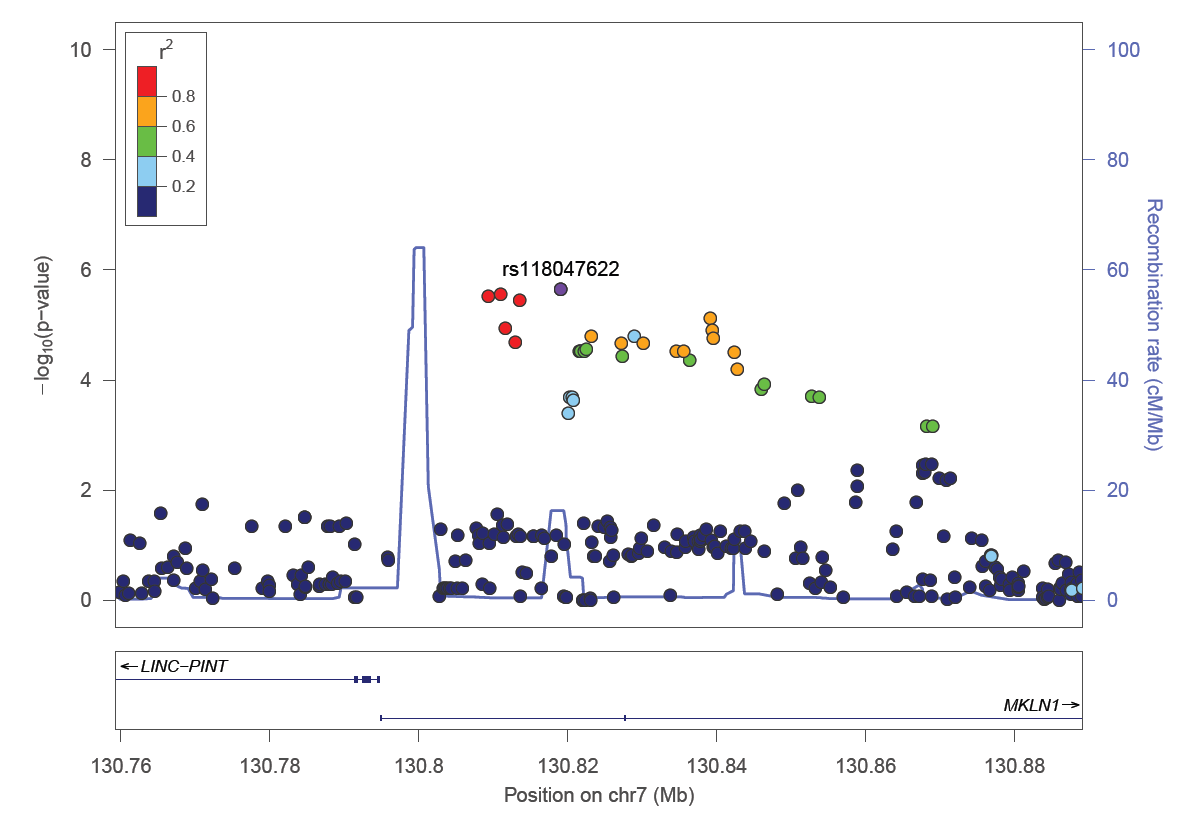
**

**Fig C**

**
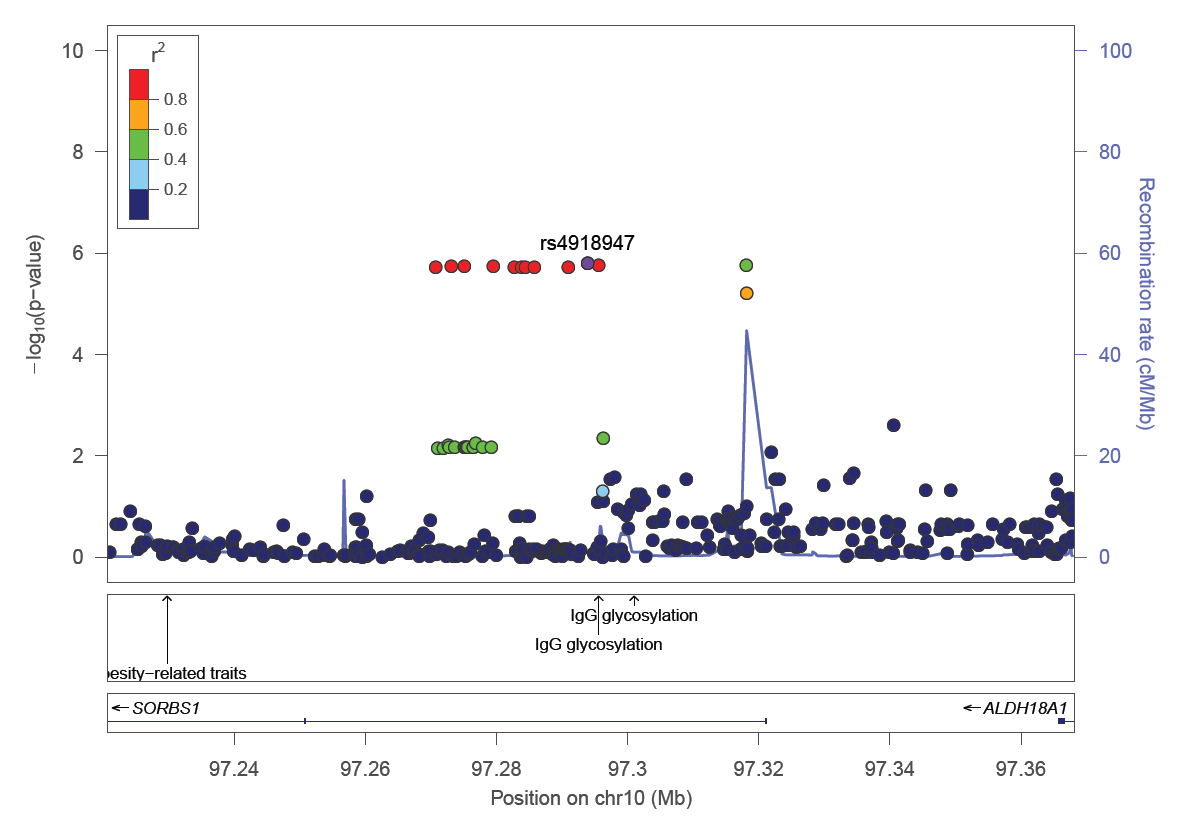
**

**Fig D**

**
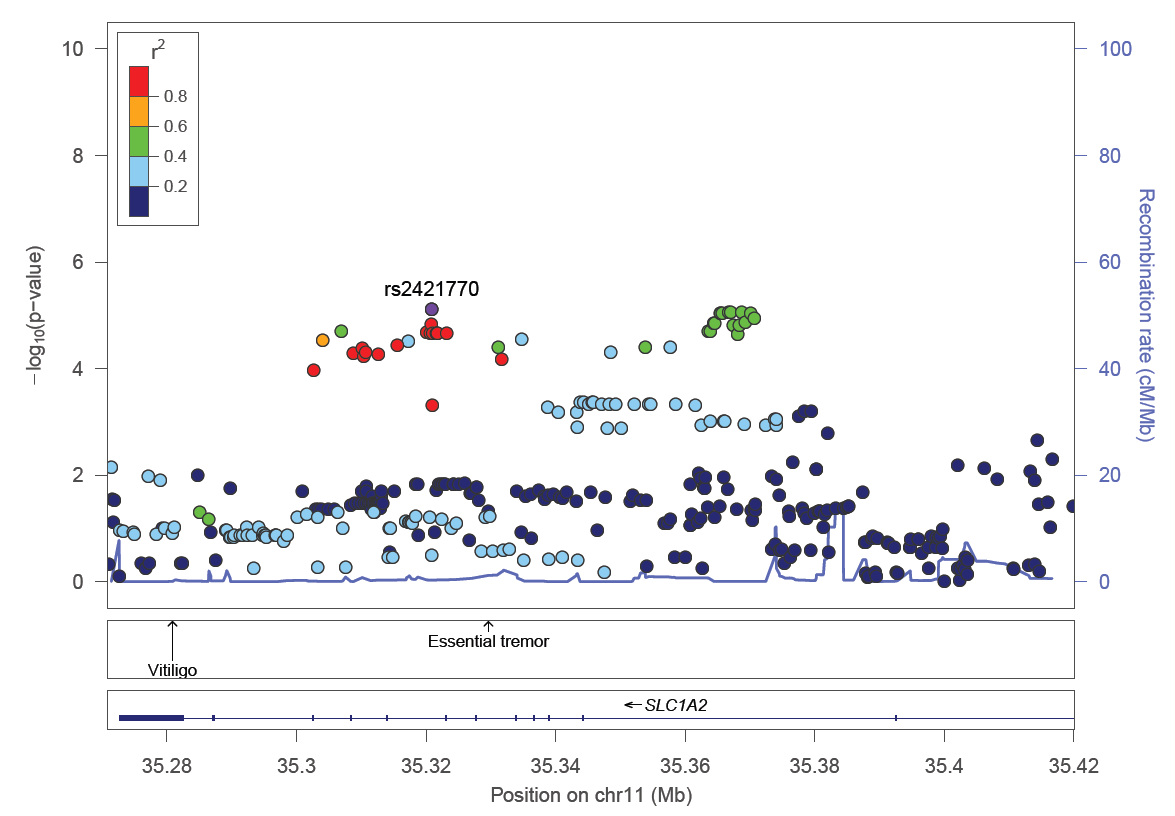
**

**Fig E**

**
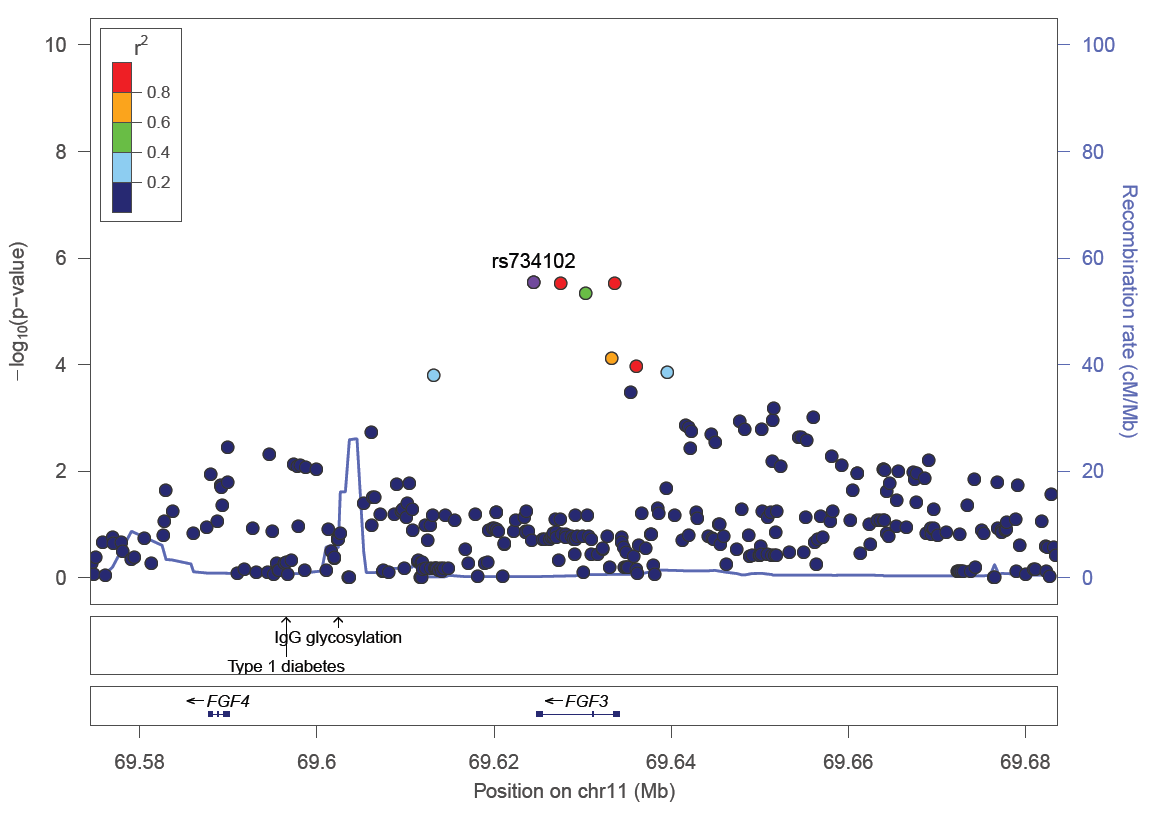
**

**Fig F**

**
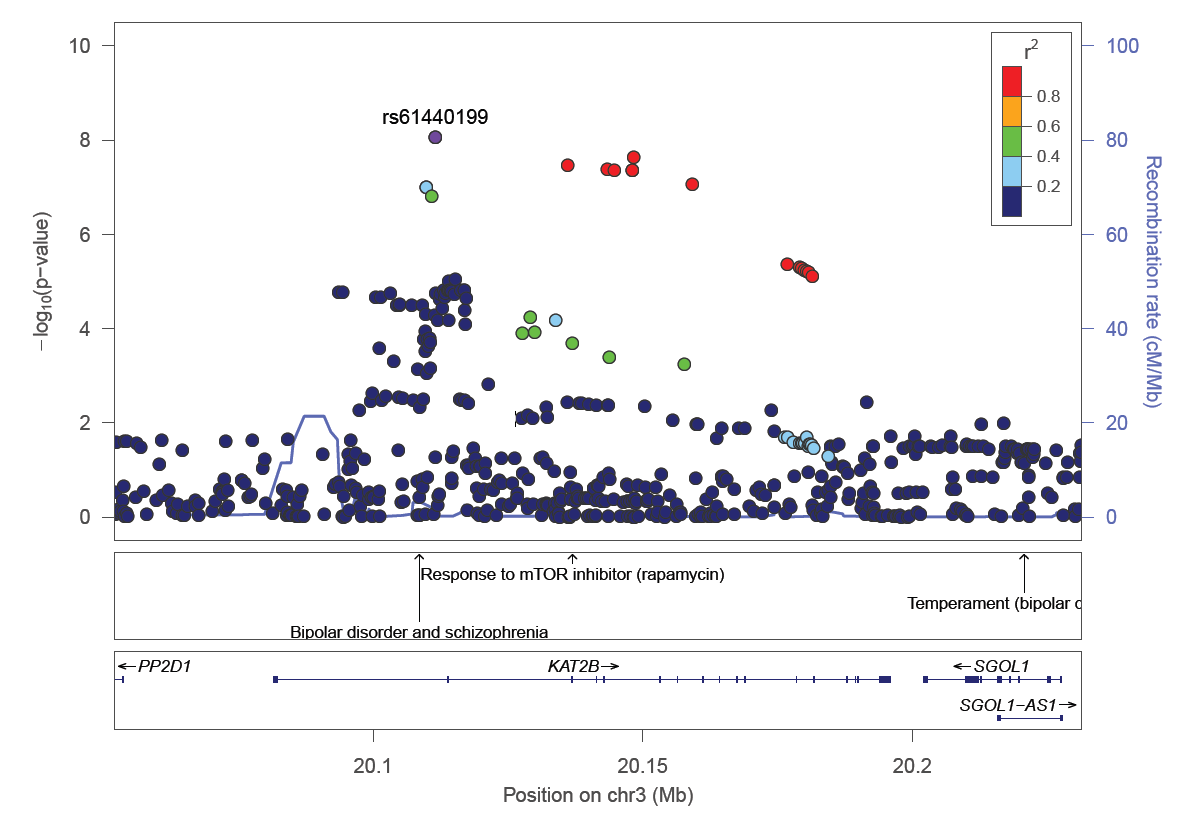
**

**Fig G**

**
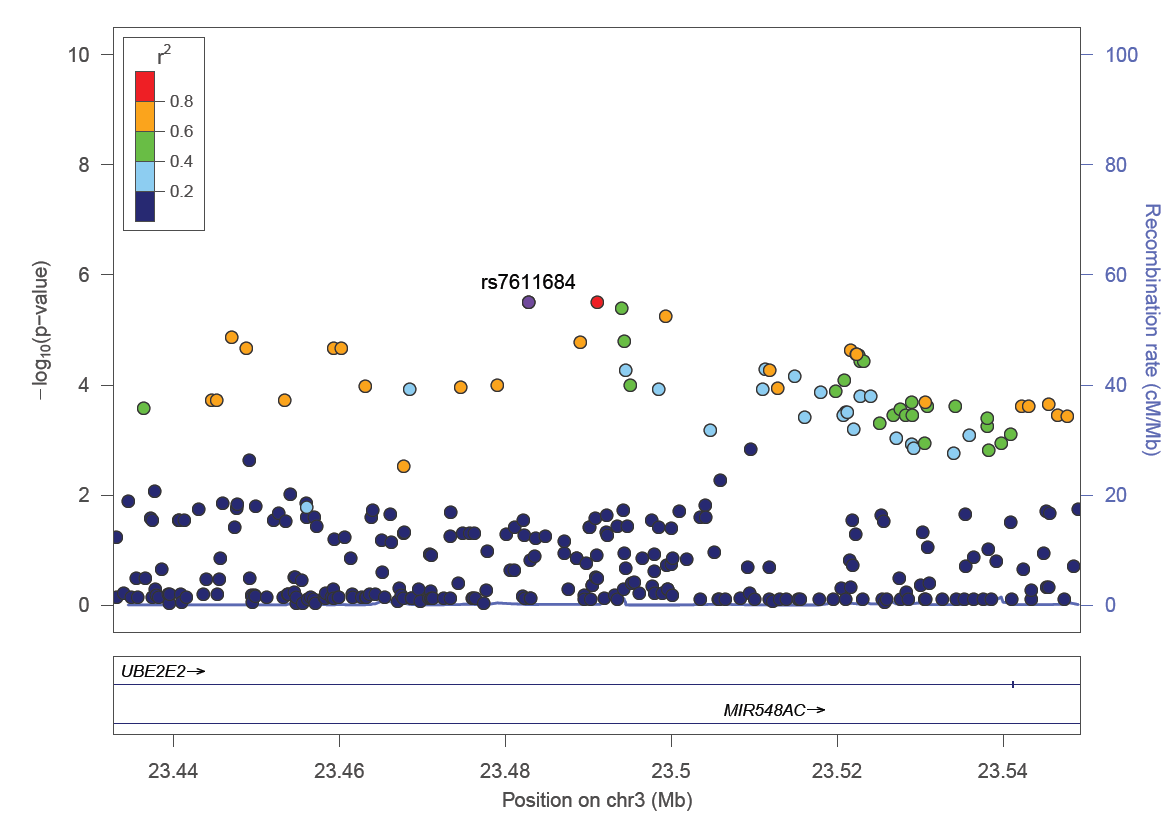
**

**Fig H**

**
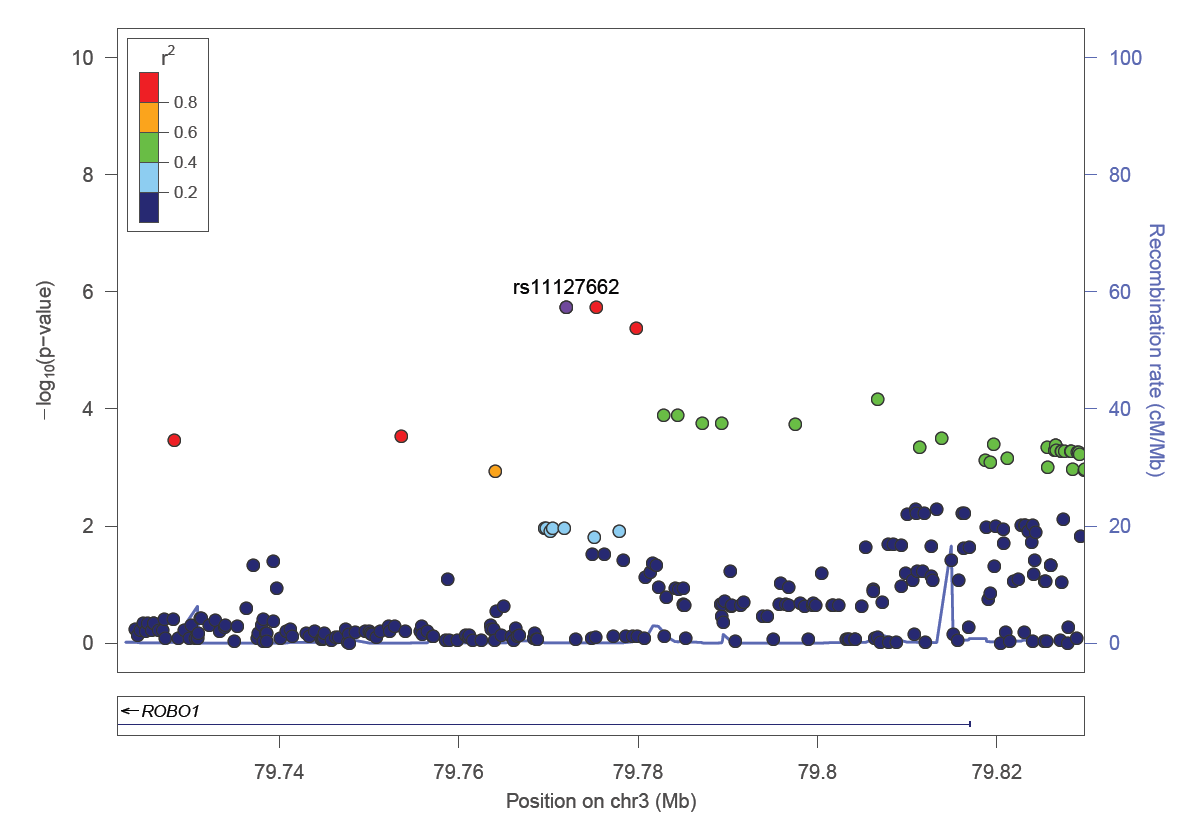
**

**Fig I**

**
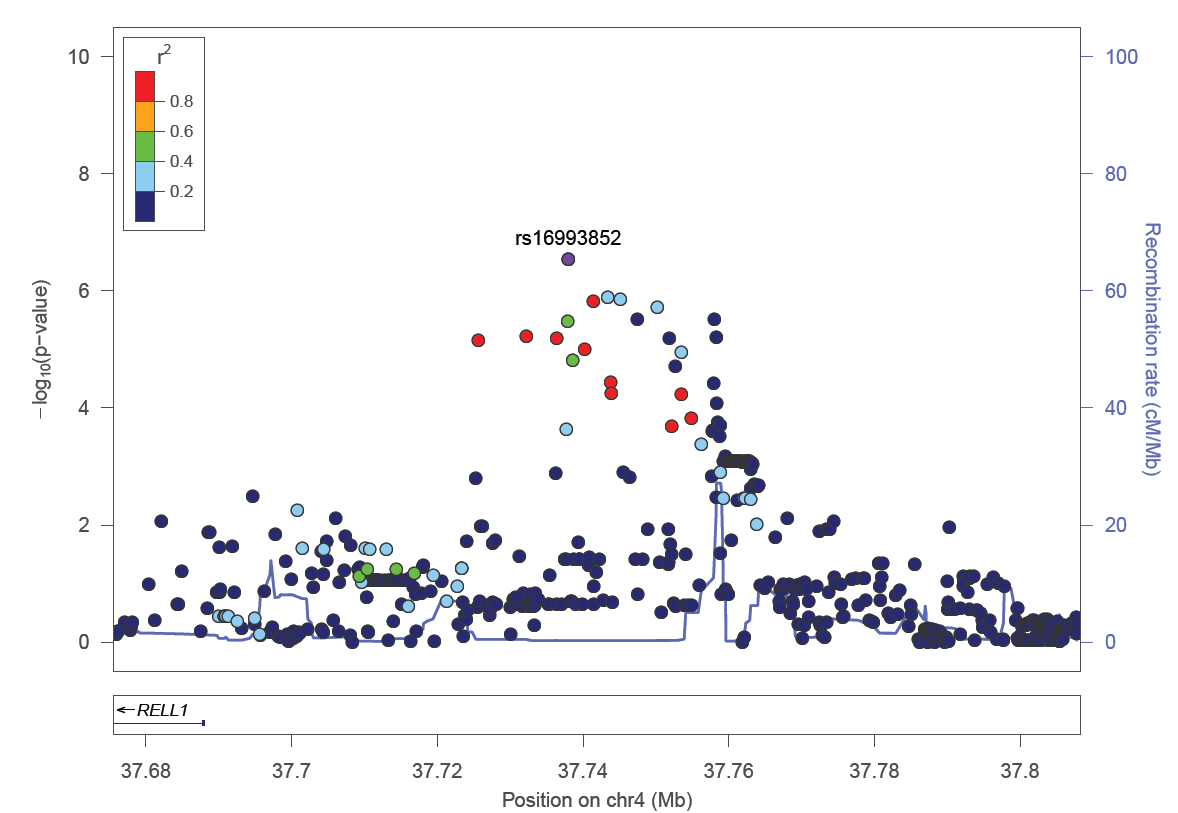
**

**Fig J**

**
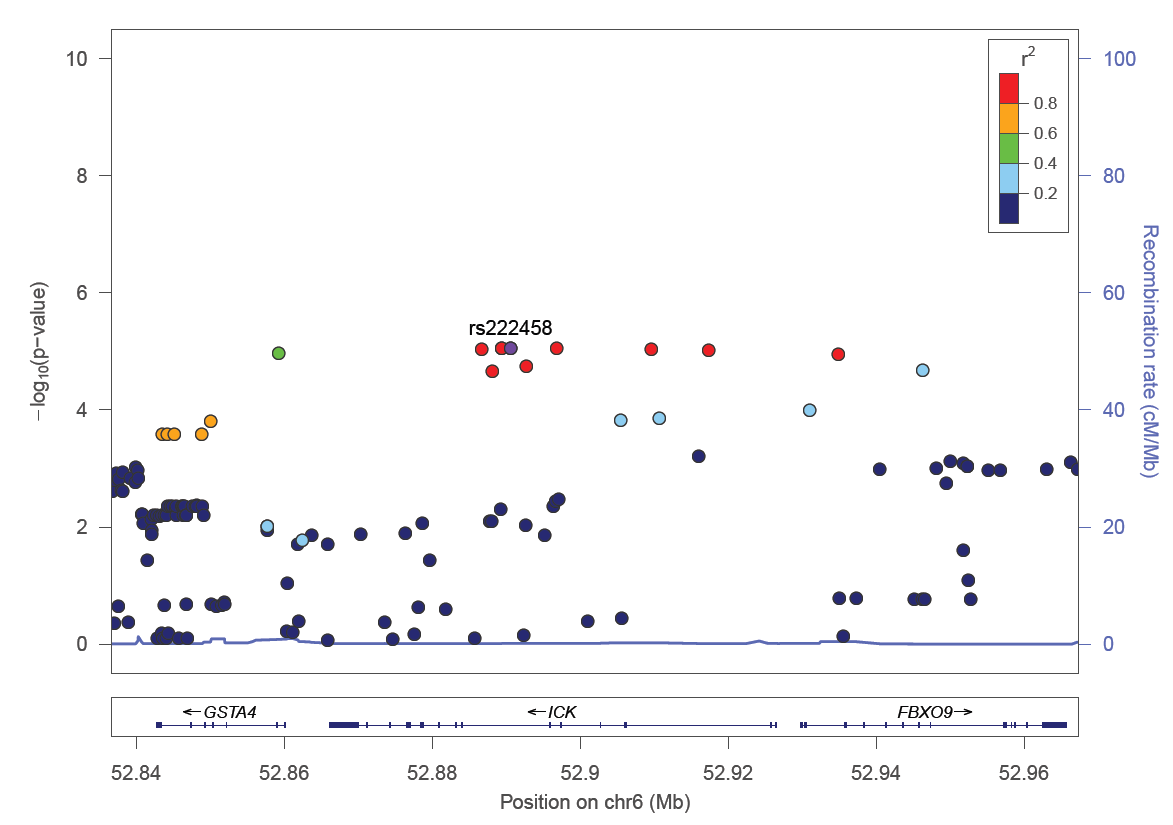
**

**Fig K**

**
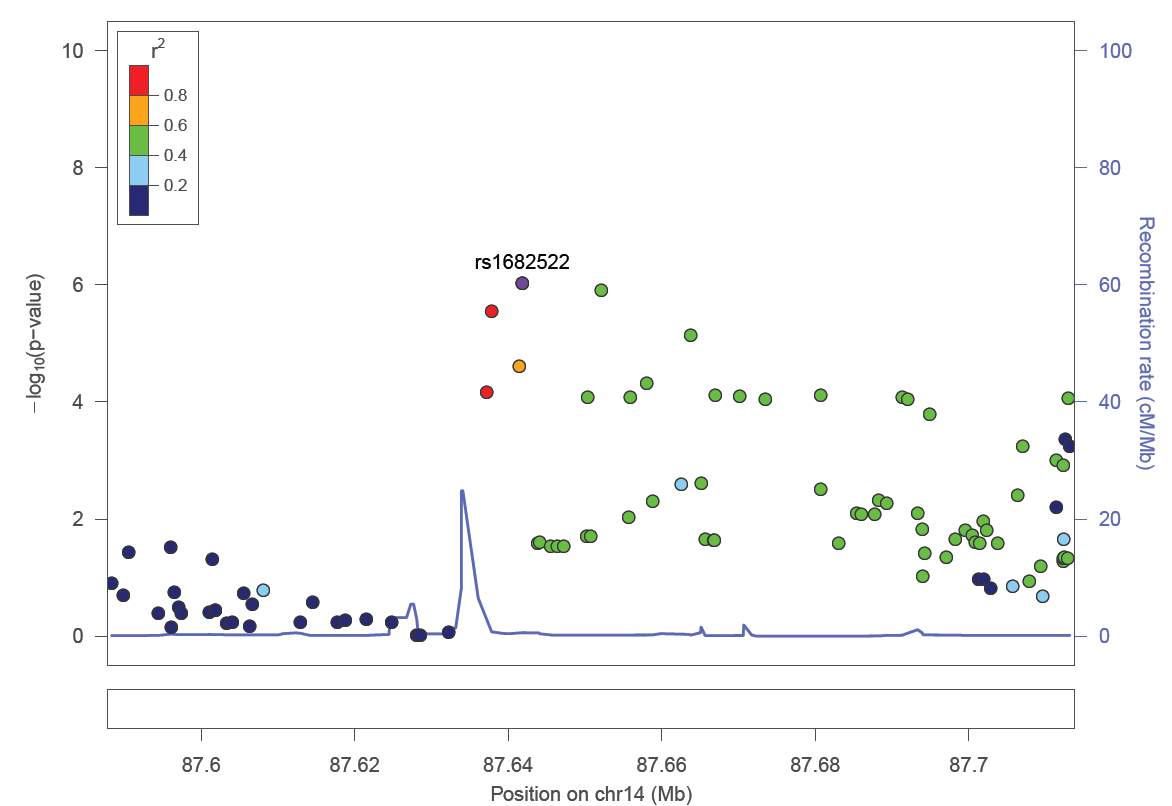
**

**Fig L**

**
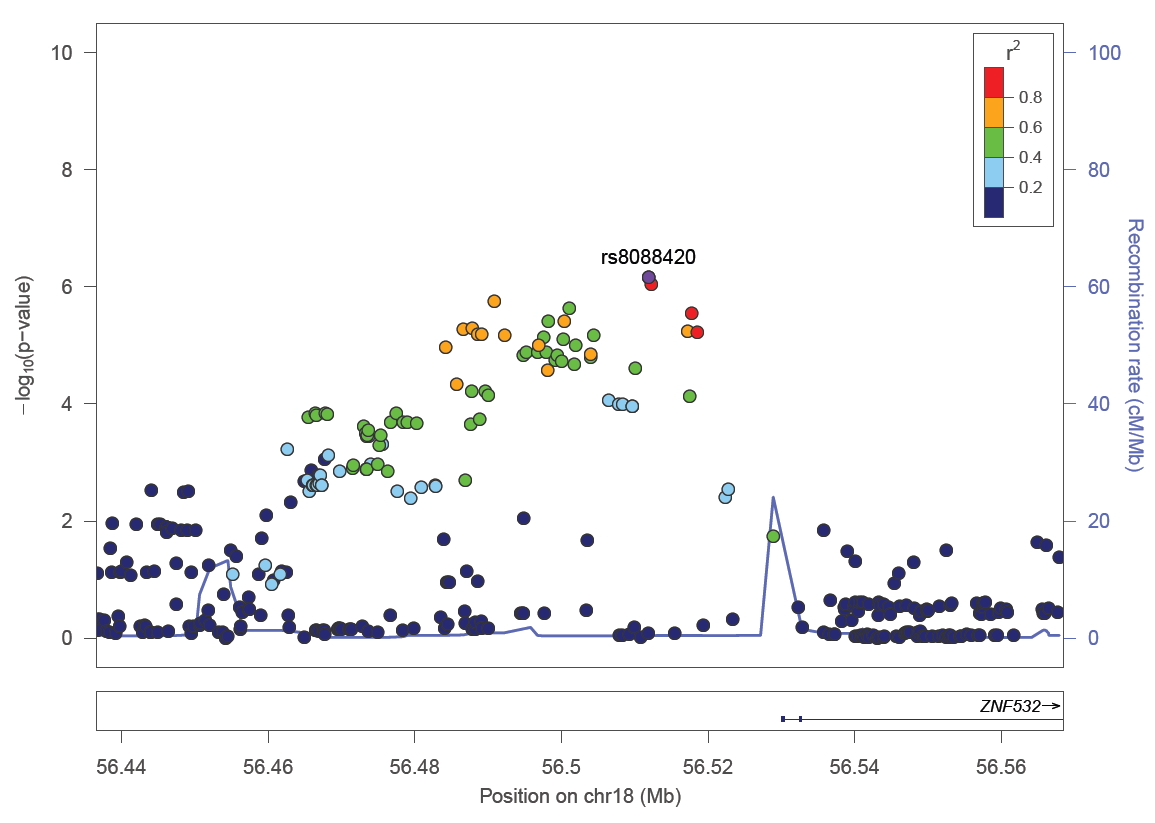
**

**Fig M**

**
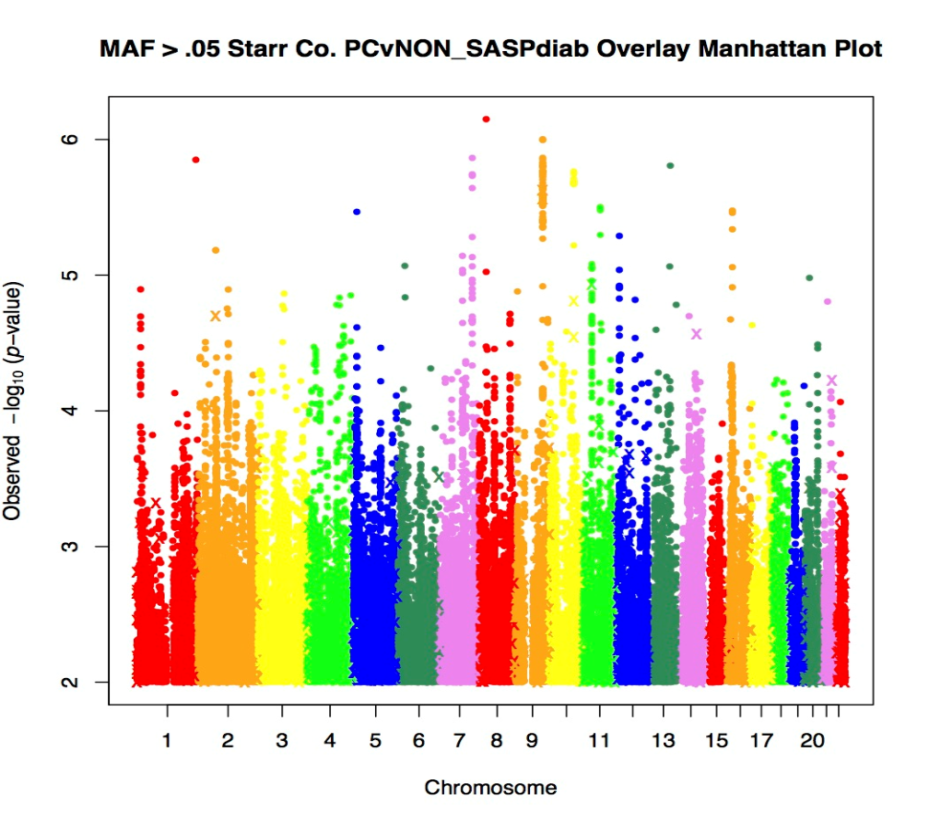

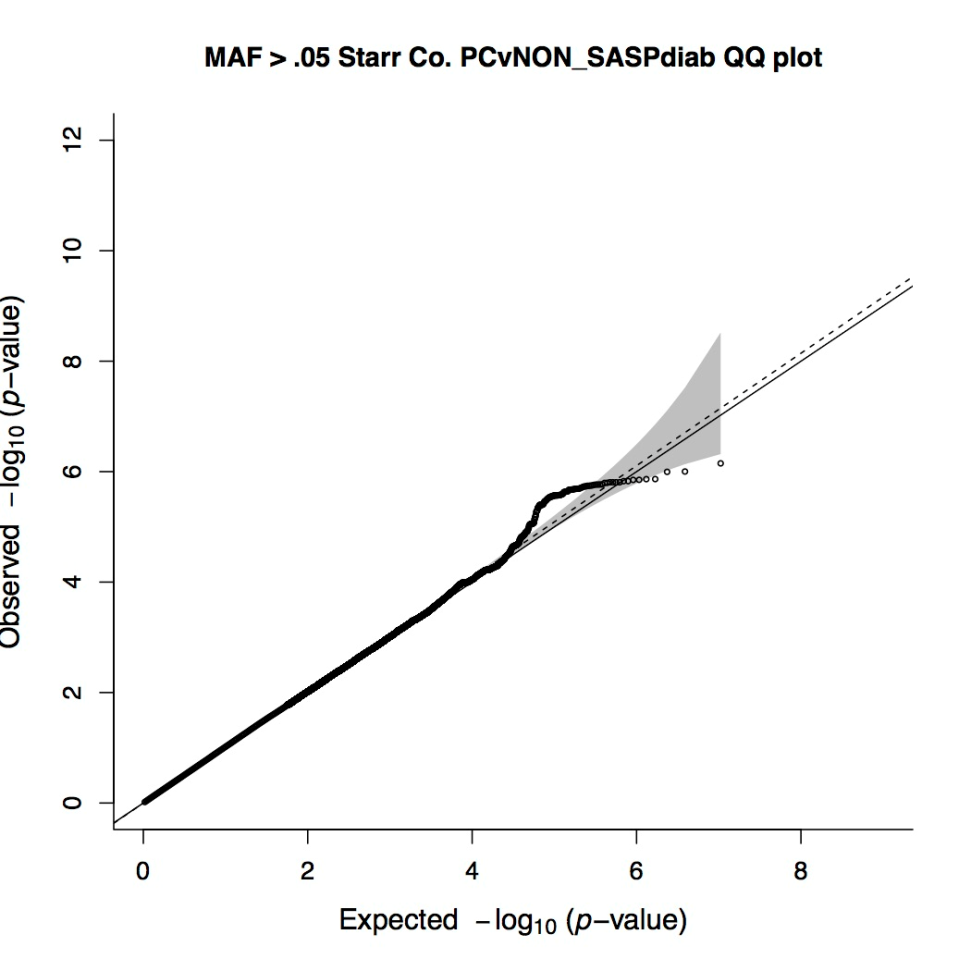
**

**Fig N**

**
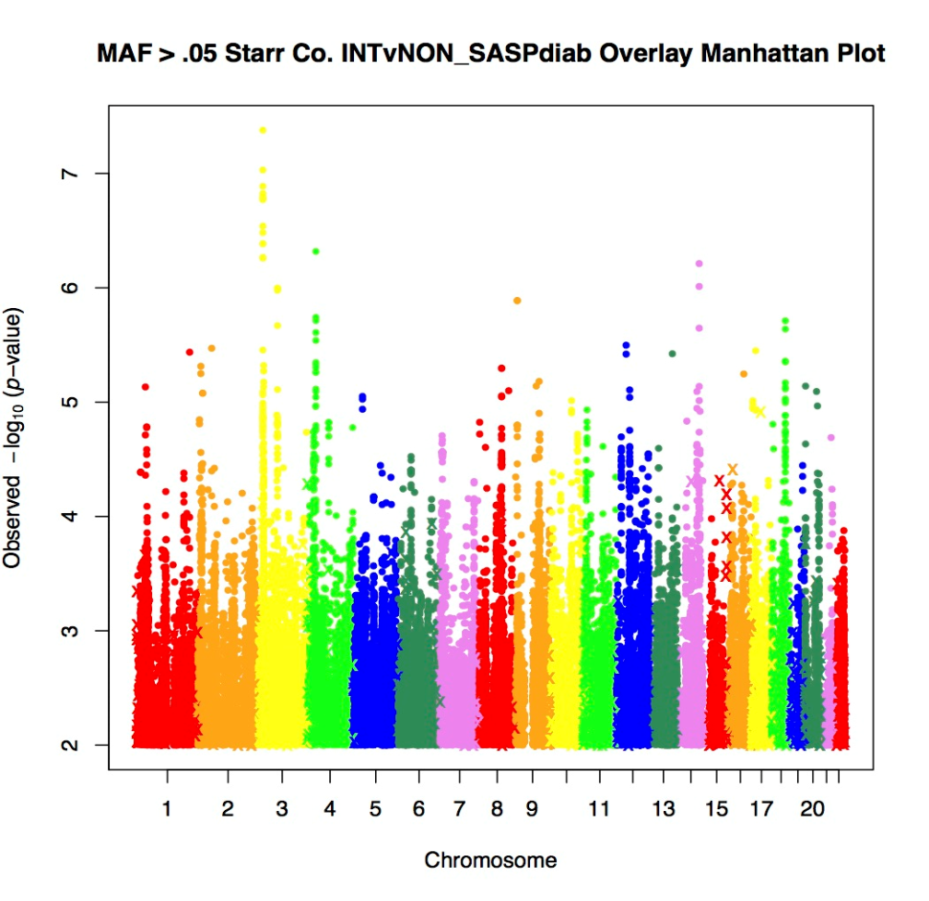
**


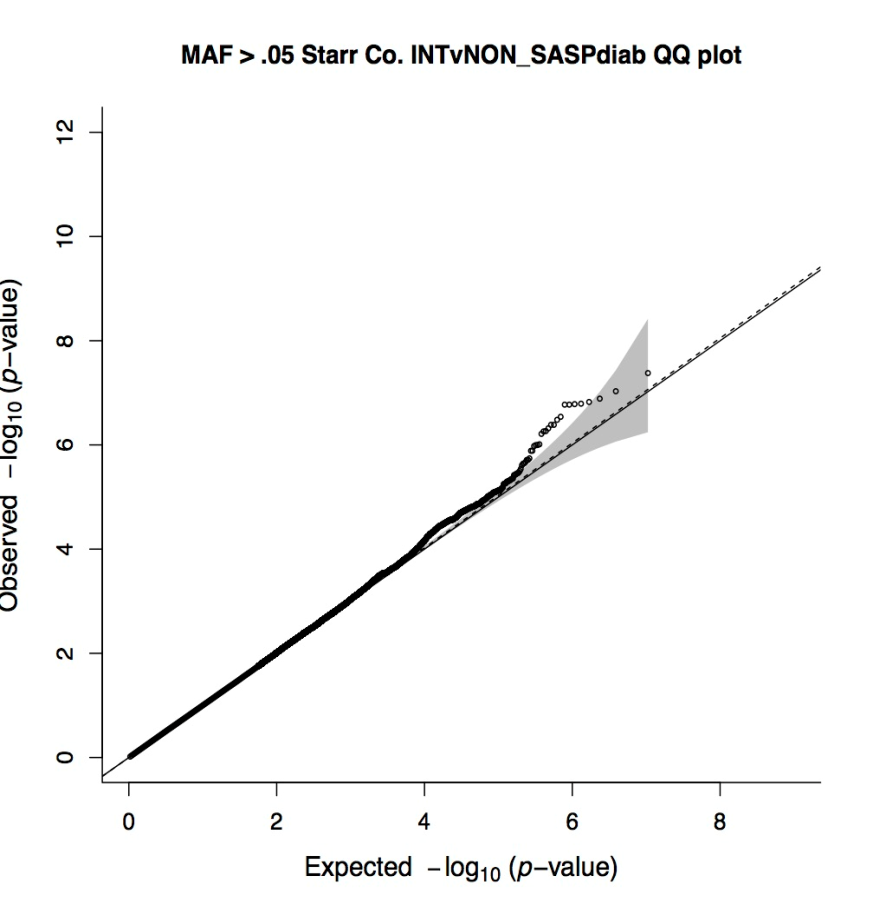


**Fig O**

**
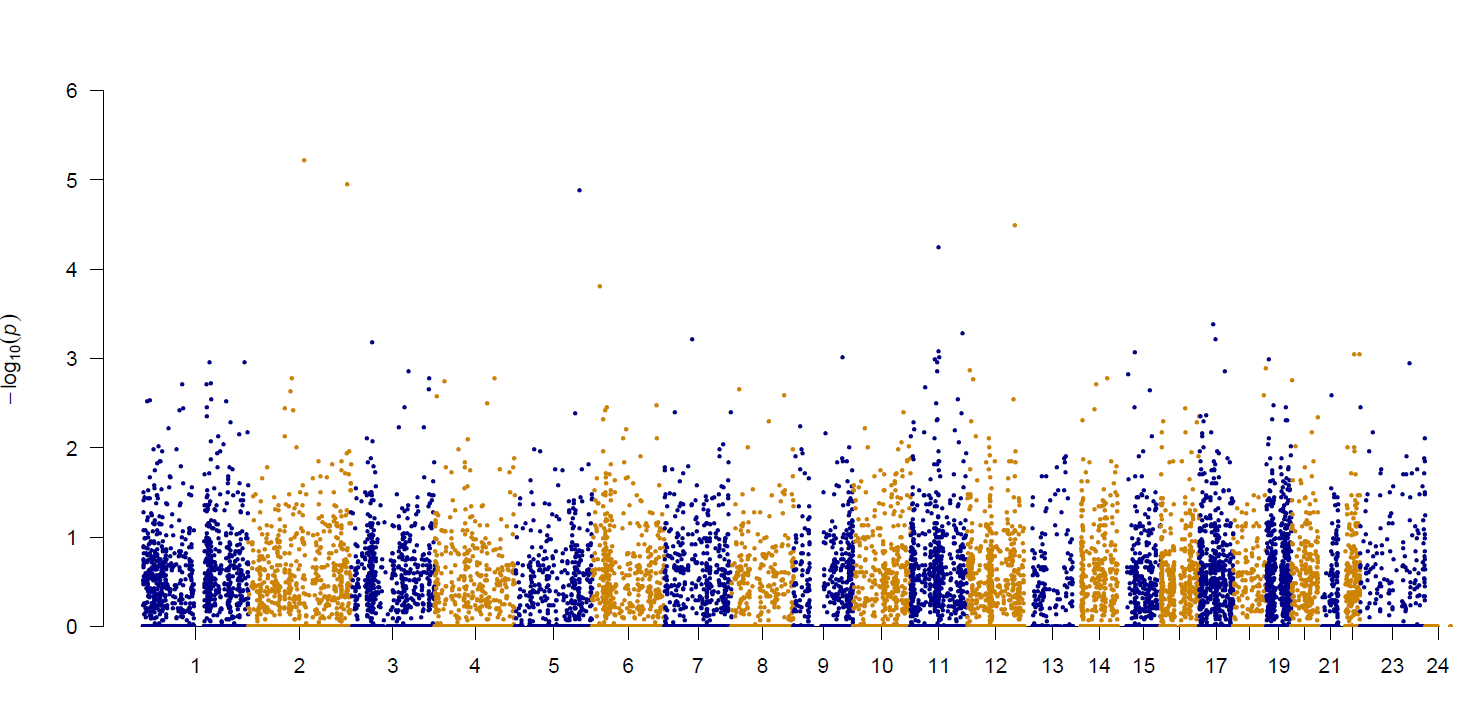
**

**
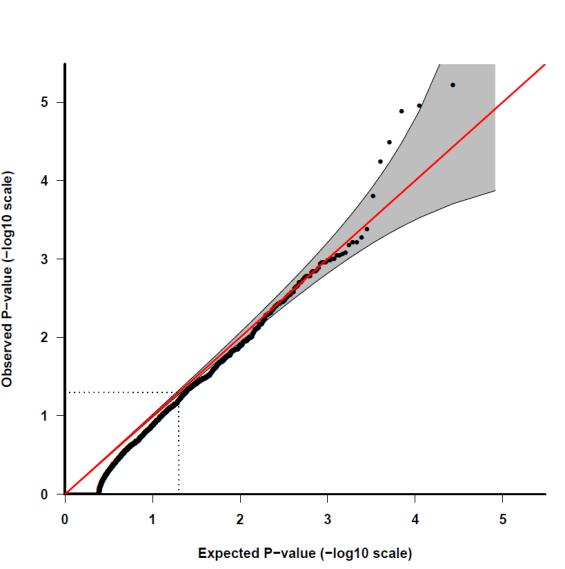
Fig P
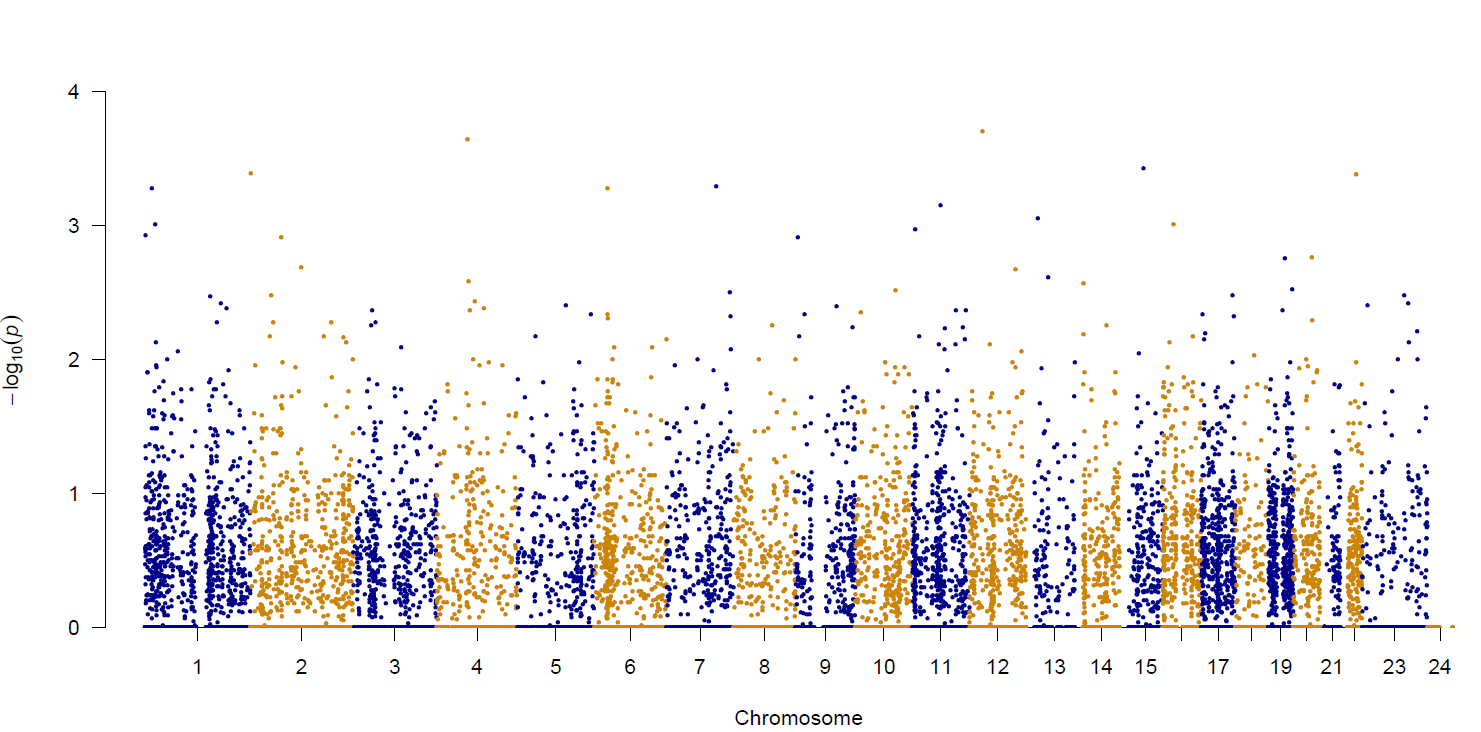
**

**
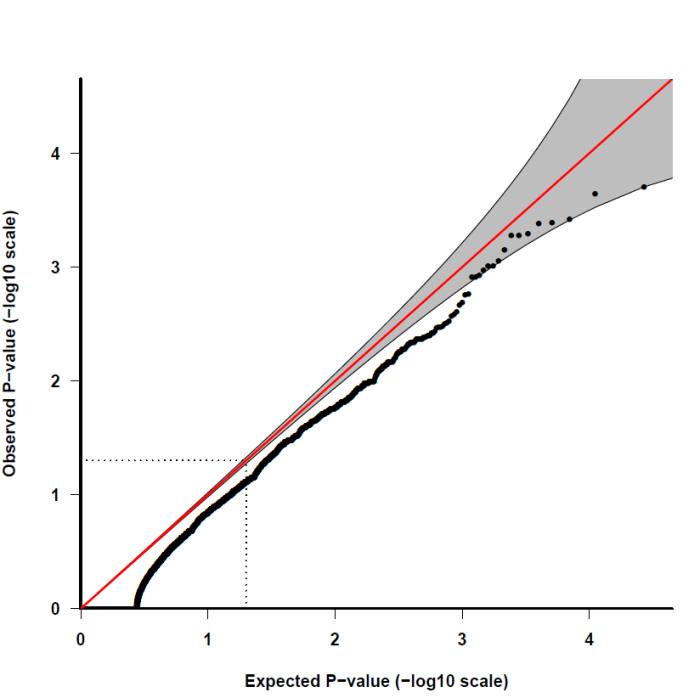
Fig Q**

**
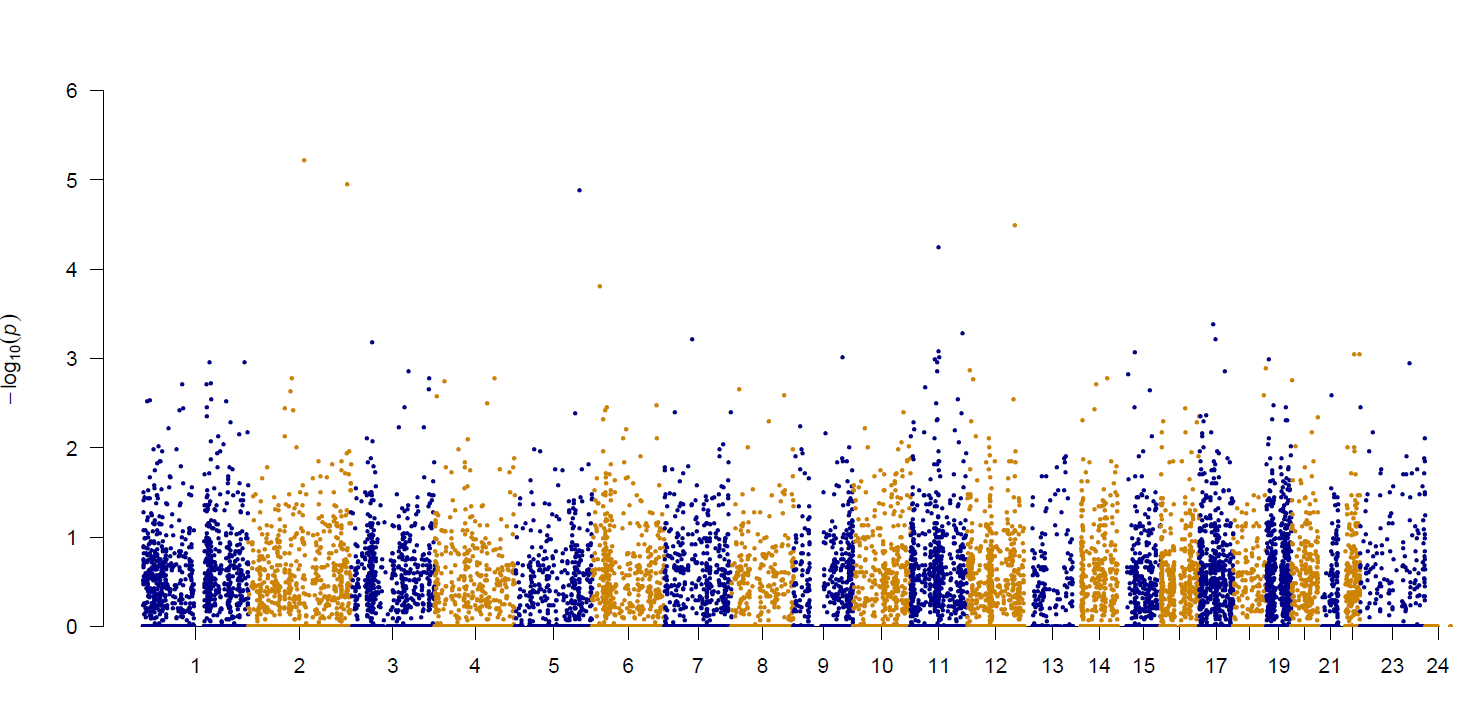
**

**
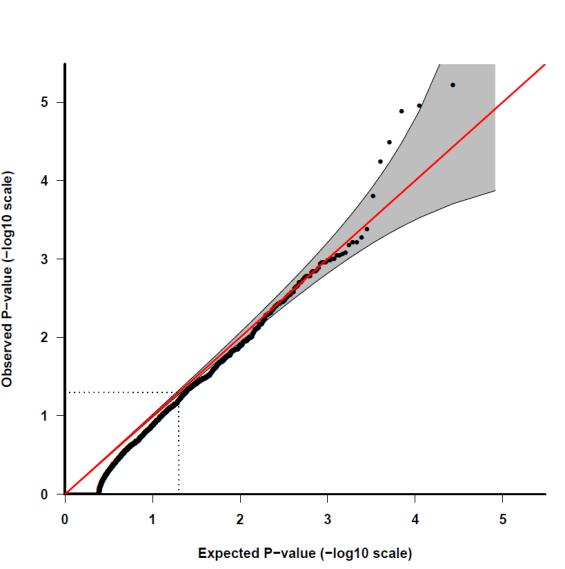
Fig R**

**
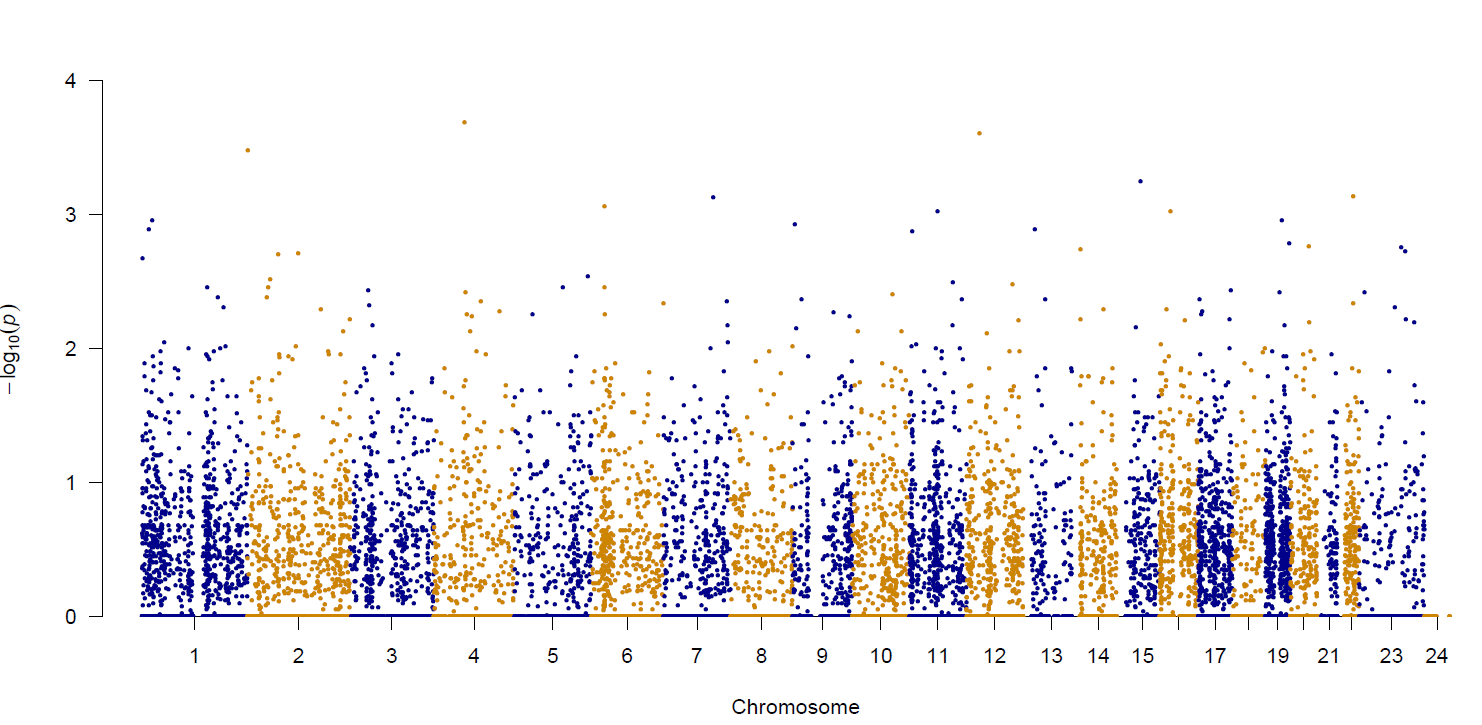

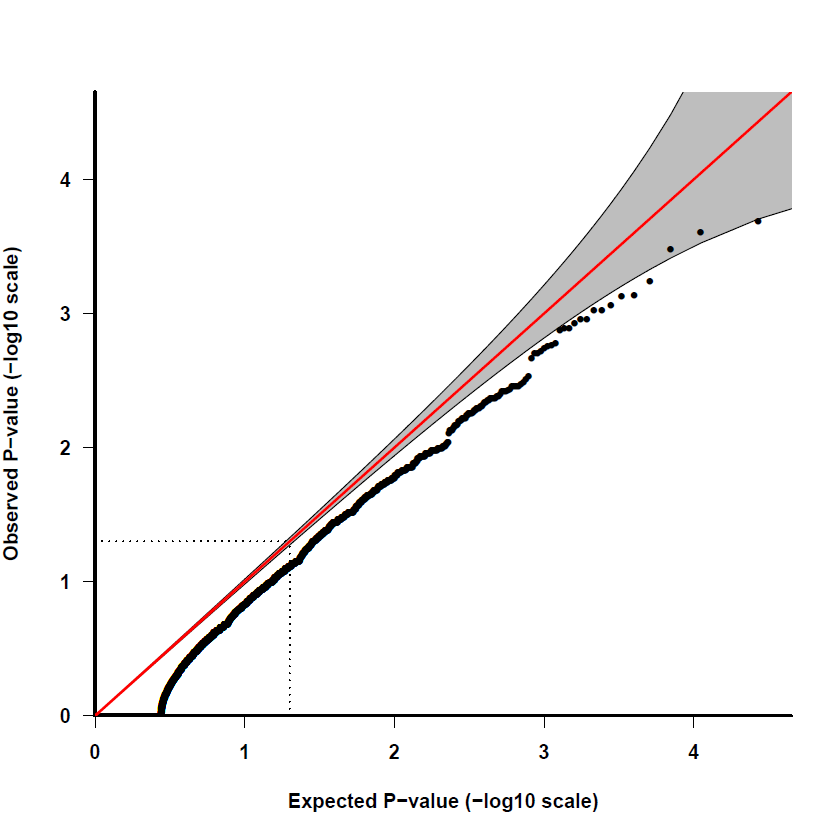
Fig S**

**
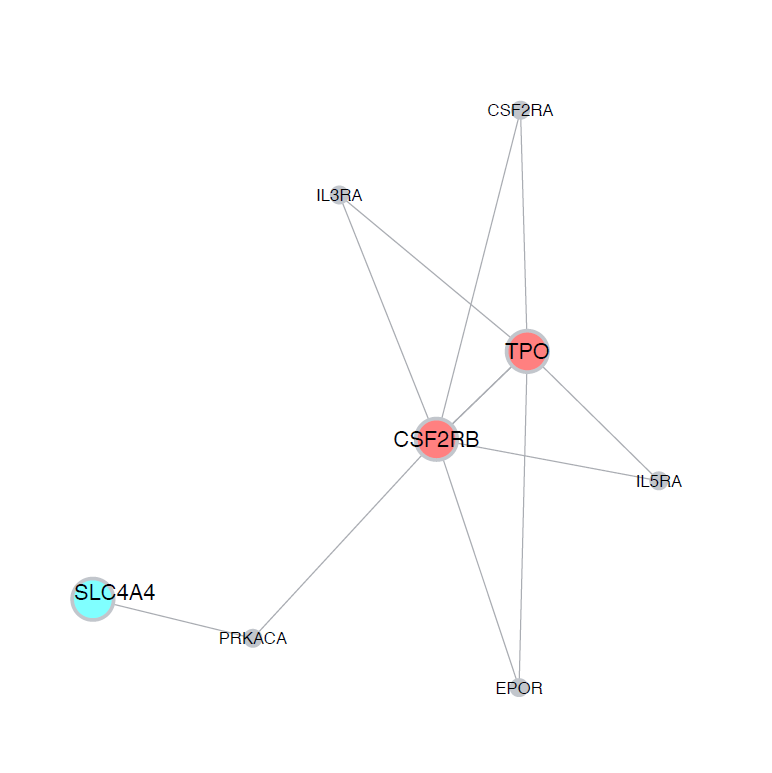
**
